# Characterization of Phase I and Glucuronide Phase II Metabolites of 17 Mycotoxins Using Liquid Chromatography—High-Resolution Mass Spectrometry

**DOI:** 10.3390/toxins11080433

**Published:** 2019-07-24

**Authors:** Irina Slobodchikova, Reajean Sivakumar, Md Samiur Rahman, Dajana Vuckovic

**Affiliations:** 1 Department of Chemistry and Biochemistry, Concordia University, 7141 Sherbrooke Street West, Montreal, QC H4B 1R6, Canada; 2 Centre for Biological Applications of Mass Spectrometry, Concordia University, 7141 Sherbrooke Street West, Montreal, QC H4B 1R6, Canada

**Keywords:** mycotoxins, metabolism, high-resolution mass spectrometry (HRMS), microsomal incubation, glucuronidation, human, biomonitoring

## Abstract

Routine mycotoxin biomonitoring methods do not include many mycotoxin phase I and phase II metabolites, which may significantly underestimate mycotoxin exposure especially for heavily metabolized mycotoxins. Additional research efforts are also needed to measure metabolites in vivo after exposure and to establish which mycotoxin metabolites should be prioritized for the inclusion during large-scale biomonitoring efforts. The objective of this study was to perform human in vitro microsomal incubations of 17 mycotoxins and systematically characterize all resulting metabolites using liquid chromatography–high-resolution mass spectrometry (LC-HRMS). The results obtained were then used to build a comprehensive LC-MS library and expand a validated 17-mycotoxin method for exposure monitoring to screening of additional 188 metabolites, including 100 metabolites reported for the first time. The final method represents one of the most comprehensive LC-HRMS methods for mycotoxin biomonitoring or metabolism/fate studies.

## 1. Introduction

Mycotoxins are toxic chemically diverse secondary metabolites produced by filamentous fungi. Their structural diversity can give rise to several adverse effects in humans and animals, such as carcinogenicity, immunosuppression, teratogenicity, nephrotoxicity, and hepatotoxicity [1]. The contamination of food and feed supply with low levels of mycotoxins is widespread, and includes commodities such as wine, apple juice, cereals, milk, coffee beans, maize, nuts, dried fruits, and meat products [2,3,4,5,6,7]. For example, a worldwide survey of more than 19,000 cereal and oilseed samples found that 72% were contaminated with one or more mycotoxins: aflatoxins (26%), deoxynivalenol (56%), ochratoxin A (25%), fumonisins (54%), and zearalenone (37%) [8]. In agreement with these findings, the most recent surveys of Canadian food supply showed 59% and 75% of the tested samples had detectable levels of at least one mycotoxin, with the most frequent incidence of deoxynivalenol [2,9]. Many other studies to date have also confirmed co-occurrence of multiple mycotoxins in food and feed samples [8,10,11], which in turn may lead to synergistic or antagonistic effects. Currently, the assessment of human mycotoxin exposure is primarily modelled from the measured/estimated levels of mycotoxins in the various foods and the calculated daily average food intake of various food groups to estimate population exposure and introduce regulations for food monitoring when appropriate. However, an individual’s food consumption pattern depends on personal preferences. Thus, population-based food intake models can lead to the inaccurate estimation of human exposure to mycotoxins and, subsequently, higher health risk in some sub-populations. Vegetarian and non-vegetarian adult exposure to deoxynivalenol is one such example, whereby a recent U.K. study found ~2 × higher mean level of deoxynivalenol in vegetarians than in non-vegetarians [12]. Furthermore, the exceeded recommended tolerable daily intakes (32%) were found only in individuals belonging to the vegetarian group. Biomonitoring of adult and children urine samples in large-scale exposure studies also demonstrated that daily tolerable intake was exceeded for some mycotoxins [13,14,15]. 

To address the limitations of food-based models, direct human biomonitoring of biological fluids is proposed as an alternative approach to assess health risk [13,14,15]. However, this approach currently has several limitations. It requires high-throughout, multi-mycotoxin methods that have very low limits-of-detection in complex biological matrices such as blood and urine. Secondly, metabolic pathways have not been investigated thoroughly for all mycotoxins and key metabolites have not yet been prioritized for inclusion in routine biomonitoring [16]. Consequently, most of the existing analytical LC-MS methods used for the assessment of human exposure focus only on the detection of parent compounds. This can lead to significant underestimation of mycotoxin exposure. For example, a recent study of deoxynivalenol metabolism in humans confirmed the need for the inclusion of its metabolites in biomonitoring [17]. They showed that approximately 72.6% of total urinary deoxynivalenol (DON) was composed of its glucuronides, DON-15-glucuronide (15-Gluc-DON) and DON-3-glucuronide (3-Gluc-DON) and only 27.4% was present as free DON [17]. Thus, the measurement of DON only would underestimate DON exposure by ~4 ×. Other studies have also confirmed the importance of 15-Gluc-DON as a predominant glucuronide [15,18,19]. In general, biomonitoring methods should combine parent compounds and their predominant metabolite(s) in order to properly estimate exposure risk [20]. 

Currently, the majority of mycotoxin biomonitoring is performed using urine since it is non-invasive and accessible in relatively large volume. These methods can be divided into methods with and without β-glucuronidase treatment. B-glucuronidase catalyzes hydrolysis of conjugated mycotoxins, such as sulfate and glucuronide conjugates. Thus, the use of enzymatic hydrolysis can provide an appropriate alternative to direct metabolite monitoring for at least those mycotoxins which are predominately metabolized to phase II conjugated forms such as DON [17,21]. To date, such methods cover 7–11 mycotoxins [14,21,22,23]. The main disadvantages of β-glucuronidase treatment are: increases the cost per sample, requires longer processing time of about 16–18 h, and the additional step in sample preparation may give a rise to quantification errors. Multi-mycotoxin methods without β-glucuronidase treatment have been developed for 8–32 mycotoxins in urine [13,24,25,26,27,28], and for 8–27 mycotoxins in blood, serum or plasma [23,24,29,30]. However, these methods often include no or limited direct monitoring of mycotoxin metabolites.

Due to their toxicity, in vivo data on mycotoxin metabolism in humans after exposure is rare, with few exceptions [17]. Animal models have been used more frequently, but the interspecies differences in mycotoxin metabolism should be taken into consideration [31,32,33]. In vitro human liver microsomal incubations have been used extensively in the metabolism studies of mycotoxins, for example to obtain metabolic profile of T-2 and HT-2 toxins [32,33], or to study in detail the glucuronidation of the zearalenone group [34,35,36]. Human liver microsomes contain a variety of enzymes that are involved in both phase I and phase II toxin metabolism and reaction conditions can be easily controlled to generate the needed quantity of metabolites. The examples of phase I reactions are oxidation, reduction, dehalogenation, or hydrolysis and are catalyzed by several enzymes including cytochrome P450. Phase II reactions are conjugation reactions, for example with glucuronic acid, sulfate, glutathione and/or amino acids. High-resolution mass spectrometry (HRMS) provides an excellent analytical platform for the characterization and investigation of mycotoxin metabolites and putative biomarkers for further human biomonitoring [20]. The combination of HRMS and metabolic software can greatly speed up and expand the ability to capture the broad spectrum of mycotoxin metabolites using both accurate-mass of full-scan MS and/or fragmentation mass spectral data (MS/MS or MS^n^). For instance, Yang et al. used HRMS to study T-2 and HT-2 metabolism in different species and identify main metabolic pathways and novel metabolites [32,33]. However, such single-analyte metabolism studies relied on a variety of analytical platforms and methods, thus hindering the creation of a comprehensive metabolite LC-MS library using a single analytical method and its further application in human biomonitoring. As such, it is of utmost importance to include mycotoxin metabolites in ongoing biomonitoring efforts and to use this information to prioritize the most commonly observed mycotoxin metabolites that may contribute to under-estimation of exposure. To achieve this goal, the first step is to fully characterize and build a comprehensive LC-MS library of mycotoxin metabolites using a single well-characterized LC-MS method.

In this work we present in vitro metabolism studies of 17 mycotoxins detected in the Canadian food supply: aflatoxins B1, B2, G1 and G2 (AFB1, AFB2, AFG1, AFG2), zearalenone (ZEN), 7-α-hydroxy-zearalenol (α-ZOL), 7-β-hydroxy-zearalenol (β-ZOL), zearalanone (ZAN), 7-α-hydroxy-zearalanol (α-ZAL), 7-β-hydroxy-zearalanol (β-ZAL), T-2 toxin (T-2), HT-2 toxin (HT-2), DON, nivalenol (NIV), 15-acetyldeoxynivalenol (15-AcDON), 3-acetyldeoxynivalenol (3-AcDON) and fusarenon X (FUS-X) in order to characterize phase I and glucuronide phase II mycotoxin metabolites. Mycotoxin metabolites were generated in vitro using pooled human liver microsomes to build an extensive in-house library of these species, for which standard compounds are often not commercially available. The final in-house LC-MS library was built using a previously published validated method for sensitive quantitation of 17 mycotoxins in plasma using liquid chromatography–high-resolution mass spectrometry (LC-HRMS) [29]. The use of this method allowed excellent chromatographic separation of many isomers and the optimized highly sensitive HRMS detection allowed detailed characterization of both known and novel metabolites.

## 2. Results

Human liver microsomes are important and common tool for in vitro investigations of toxin metabolism because they express a variety of enzymes which are involved in phase I metabolism such as microsomal cytochrome P450 (P450) and flavin-containing monooxygenases (FMO). These enzymes are responsible for the most common phase I reactions, such as oxidation. Usually, toxins are converted to more polar compounds due to phase I reactions. Phase II metabolism provides an additional mechanism to clear toxins from the body by adding water-soluble groups, such as glucuronic, methyl, sulfate and acetyl groups [20,37]. In this work, phase II glucuronidation reaction was chosen as a major human metabolic pathway of toxins in addition to phase I metabolism. In total, 17 mycotoxins, such as trichothecene type A (T-2 and HT-2), trichothecene type B (NIV, FUS-X, DON, 3-AcDON and 15-AcDON), aflatoxins (AFB1, AFB2, AFG1, and AFG2) and group of zearalenone (ZEN, α-ZOL, β-ZOL, ZAN, α-ZAL, and β-ZAL) were incubated individually in the presence of human microsomes and 188 different fungal metabolites were characterized and detected. The analysis of all microsomal incubation samples was performed with high-resolution mass spectrometer (LTQ Orbitrap Velos) coupled with liquid chromatography in order to detect and identify mycotoxin metabolites. Structural elucidation of metabolites was performed using data-dependent MS/MS acquisition and collision-induced dissociation (CID) fragmentation technique. Metabolite characterization and data analysis was performed using Compound Discoverer software 2.1, which contains extensive libraries of common metabolic pathways and mass spectral libraries.

**Figure 1:**
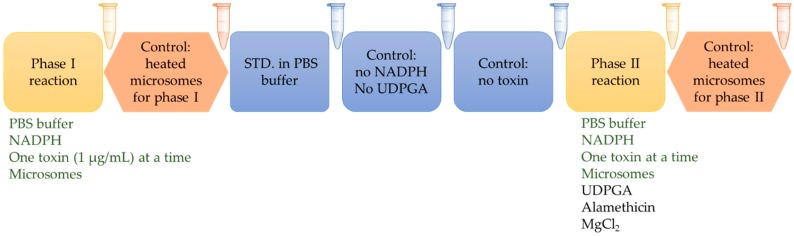
Scheme of microsomal incubation experiment to generate phase I and glucuronide phase II metabolites.

**Figure 2:**
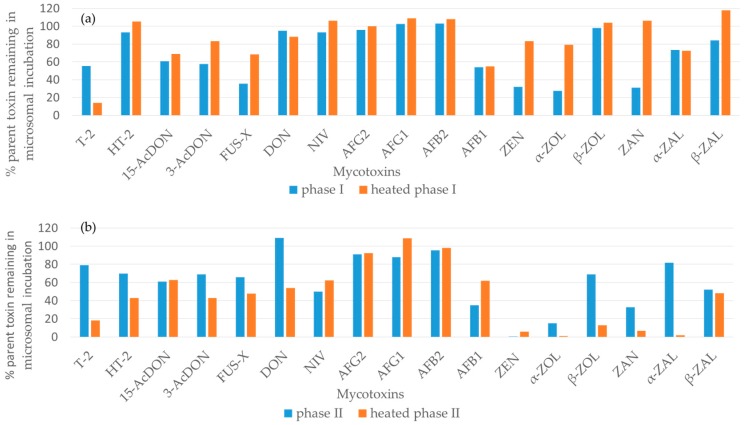
Comparison of the amount of parent toxin remaining after incubation in phase I and heated phase I (**a**) and phase II and heated phase II (**b**) microsomal incubation samples.

To confirm enzymatic origin of metabolites, besides test samples for the phase I and II, several controls were used as shown in Figure 1 and Supplementary Table S1: standard that contains toxin dissolved in PBS buffer, control without any co-factors, control without NADPH, but with UDPGA, and controls with previously heated microsomes (45 °C) for both phase I and II samples. The mycotoxin standard control and the controls without cofactors were used to highlight and eliminate the metabolites that are not enzymatically produced from the final LC-MS library. Finally, the controls without toxin ensured that any endogenous species present in microsomes would not be misidentified as mycotoxin metabolites. The controls with pre-heated microsomes were included in the experiment to test the stability of microsomal enzymes. In heat-inactivated samples, the metabolic activity was changed and the generation of metabolites was reduced during the phase I metabolic reactions indicating that the responsible enzymes were sensitive to heat (Figure 2a). However, an opposite effect was observed in the phase II reactions, whereby an increased rate of glucuronidation was observed in all mycotoxin samples (Figure 2b). The deactivation of phase I metabolism observed in our study matches the previously published work about enzyme stability [38,39]. In contrast, uridine 5′-diphospho-glucuronosyltransferases (UGTs), key enzymes used in our phase II glucuronidation reactions, appear to be thermally stable enzymes [40], and the heat-inactivation step was beneficial to generating additional glucuronide metabolites in sufficient quantities for detailed characterization. 

### 2.1. Trichothecene Type A and B

#### 2.1.1. Trichothecene Type A

**Figure 3:**
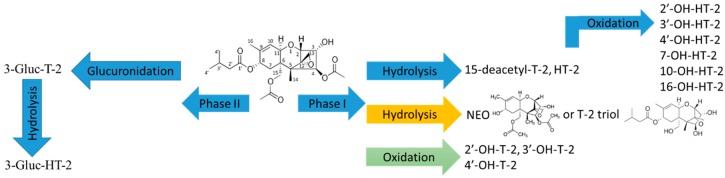
Microsomal biotransformation of T-2 toxin in phase I and phase II reactions.

The list of T-2 generated metabolites is shown in the Supplementary Table S2. There were two main pathways for T-2 metabolites, hydrolysis and oxidation in phase I (Figure 3). The chromatographic separation of T-2 and its metabolites is shown in Supplementary Figures S1 and S2. The identification of metabolites was performed by comparing [M+Na]^+^ product ion mass spectra of T-2 and its metabolites. The fragmentation pattern of T-2 showed some characteristic fragments, 387.2 m/z, 327.2 m/z and 267.2 m/z due to the loss of isovaleric acid (C_5_H_10_O_2_, 102.1 Da) at position 8, and acetic acid (CH_3_COOH) at position 15 or 4, respectively, (Supplementary Figure S3d). The extracted ion chromatogram of [M+Na]^+^ ion at 447.1989 m/z revealed two peaks at 7.93 min (447.1988, 0.22 ppm) and 8.22 min (447.1986, 0.67 ppm), indicating the presence of two metabolites that were 42.0 Da less then T-2 (Supplementary Figures S1 and S4a,b). The peak observed at 8.22 min was identified as HT-2, since it had the same RT and MS^2^ as the authentic standard of HT2. The second peak could be putatively identified as 15-deacetyl-T-2 (15-de-Ac-T-2). 15-de-Ac-T-2 had been previously observed as a metabolite of T-2 in Wistar rats [41]. Based on the structure of T-2, the possible loss of 42.0 Da can be due to the loss of the second acetyl group at position 15. Also, both metabolites had identical MS^2^ spectra with the typical losses of isovaleric side chain (102.1 Da) and acetic acid (60.0 Da) at fragments of 345.2 Da and 285.2 Da, respectively (Supplementary Table S2 and Figure S4a,b). There was also another ion at 405.1881 (0.74 ppm) m/z corresponding to a loss of two acetyl groups from T-2, but it was very low intensity ion, so further identification was not possible (Supplementary Table S2 and Figure S1). The literature reports two possible compounds with this mass, neosolaniol and T-2 triol [41,42]. 

The second pathway observed in phase I reactions was oxidation of both T-2 and HT-2. The theoretical masses of [M+Na]^+^ ions of T-2 (505.2044) and HT-2 (463.1939) hydroxyl-metabolites were 16.0 Da higher than T-2 and HT-2, which confirmed the presence of additional oxygen in those compounds (Supplementary Table S2). There were three T-2 hydroxy metabolites observed at 6.54 min (505.2042, 0.40 ppm), 6.60 min 505.2041, 0.59 ppm), and 6.81 min (505.2043, 0.2 ppm), Supplementary Table S2 and Figure S2. All three hydroxyl metabolites had similar MS^2^ spectra, Supplementary Table S2 and Figure S3a–c). The position of hydroxyl group was identified by comparing [M+Na]^+^ product ion spectra of the hydroxyl-metabolites and T-2. In MS^2^ spectrum of hydroxyl metabolites there was a fragment with 387.2 m/z that could be generated as the loss of isovaleric acid side chain plus oxygen atom (C_5_H_10_O_3_, 118.1 Da). This fragment showed a 16.0 Da shift that indicated the position of hydroxyl group on the isovaleric side chain. Therefore, all three hydroxyl metabolites have OH group on isovaleric side at position 3′, 4′ or 2′, (Supplementary Table S2 and Figure S3a–c). Hydroxy metabolites of HT-T-2 also had a 16.0 Da shift. The numbering of HT-2 metabolites was chosen to match numbering of the peaks described for HT-2 where incubation and detailed characterization was performed with HT-2 toxin. T-2 microsomal incubation samples had 5 out of 6 metabolites of these hydroxyl metabolites, but their intensity was ~4 × less than in HT-2 incubations, Supplementary Table S2 and Figure S2. Overall, only 26% of T-2 metabolized in phase I reactions, with HT-2 as the predominant metabolite. The metabolism of T-2 has already been investigated by Yang et al. in farm animals and humans [33]. Our data are in agreement with their results, HT-2 is predominant metabolite of T-2. However, our study also generated two additional new hydroxyl metabolites of T-2. LC chromatographic separation of isomers using our pentafluorophenyl stationary phase and/or excellent limits of detection of the method may have facilitated the detection of these additional metabolites versus previous work. Furthermore, these newly detected metabolites are consistent with the available sites on T-2 for hydroxyl modifications.

In phase II reaction samples of T-2, there were two glucuronide forms, glucuronide of T-2 and HT-2 (Figure 3, Supplementary Table S2, and Figure S5). The different metabolic activity was observed in the phase II sample and heated control, about 79% and 18% of T-2 did not metabolize, respectively (Figure 2b). The most predominant glucuronide form was glucuronide of HT-2 (51%) and only 8% composed T-2 glucuronide in the heated control. Their product ion mass spectra of [M+Na]^+^, 665.2413 m/z (−0.43 ppm) and 623.2304 m/z (−0.96 ppm) showed the indicative loss for glucuronides, 176.0 Da and typical fragments, 489.2 m/z and 447.5 m/z of T-2 and HT-2 respectively, confirming the T-2 and HT-2 origin of glucuronides, Supplementary Table S2 and Figure S6a,b. Further comparison of product ion spectra of [M+NH_4_]^+^ (Supplementary Figure S6c) to literature spectra confirmed this glucuronide as 3-glucuronide-HT-2 by the presence of fragment ions of 425.2 and 499.0 and their relative intensities to each other [33,43]. Ion with 499.0 m/z should be less intense than 425.2 m/z according to published data. According to the structure of T-2 and literature data there was only one possible glucuronide of T-2 [43]. 

The generated metabolites of HT-2 are presented in Supplementary Table S3. HT-2 is a main metabolite of T-2, and has hydroxyl group at position 4 instead of acetyl group. Two pathways were observed in phase I reactions, hydrolysis and oxidation. The chromatographic separation of HT-2 and its metabolites is shown in Supplementary Figures S7 and S8. Two hydrolysis products were observed as shown in Supplementary Table S3 and Figure S7. The extracted ion of [M+Na]^+^ at m/z 363.1413 (0.28 ppm) shows the 84.1 Da mass difference from HT-2 [M+Na]^+^ ion, which can be attributed to the loss of isovaleric group at position 8 and the addition of OH group. The first peak at 3.56 min can be putatively identified as 4-deacetyl-neosolaniol (4-de-Ac-NEO) which has OH group instead of isovaleric group. The product ion spectrum of the first peak has fragments with mass of 345.1 Da and 303.1 Da that confirm the water loss and further loss of acetic acid which were also found in the product ion spectra of HT-2, Supplementary Table S3. The [M+Na]^+^ ion at m/z 463.1939 was 16.0 Da higher than HT-2 [M+Na]^+^ ion, m/z 447.1989, and confirmed the hydroxylation pathway, Supplementary Table S3. The extracted ion chromatogram displayed 6 peaks with the same m/z 463.1936 (0.65 ppm) at 5.67 min, 5.77 min, 5.95 min, 6.11 min, 6.21 min and 8.23 min, Supplementary Figure S8. The first two peaks at RTs of 5.67 min and 5.77 min have similar product ion spectra, containing an indicative fragment ion at m/z 345.5 and 345.2, respectively, Supplementary Table S3 and Figure S9a,b. These ions were generated as the loss of isovaleric side chain (C_5_H_10_O_2_) plus oxygen atom resulting in the neutral loss of 118.1 Da. The presence of these fragments confirmed the position of hydroxyl group at isovaleric side chain either at position 3′ or 4′. 3′ and 4′-hydroxy-HT-2 metabolites were observed in human and animals, respectively, by Yang et al. [33]. The third peak at 5.95 min was very low intensity, and its product ion spectra were similar to the previous peaks (Supplementary Figure S9c), assuming that OH is present at isovaleric group at position 2′. For the next three peaks, the loss of 102.1 Da results in an ion fragment with m/z 361.2 Da, so it indicates that the isovaleric side chain is not changed, Supplementary Table S3 and Figure S9d–f. Therefore, the position of OH group can be found at the position 7, 10 or 16 carbon atoms. However, the product mass spectra are similar, so further identification is not possible. Overall, six hydroxyl metabolites were also detected by Yang et al., but only four of their metabolites were observed in human liver microsomes [32]. Additionally, two peaks at RT of 5.44 min and 6.55 min were observed with the mass of 405.1880 (0.99 ppm) which corresponds to 42.0 Da difference from the parent compound (HT-2) which could indicate the loss of acyl group at position 15, Supplementary Table S3. However, their product ion mass spectra showed similar losses to HT-2. Based on the fragments at 303.2 m/z and 345.2 m/z which were generated as the loss of isovaleric acid (102 Da) and acetic acid (60 Da) respectively it was concluded that the main structure is not changed, and from the known metabolites it was not possible to propose putative structures. Overall, our data are similar to the previous metabolism studies done by Yang et al. [32], confirming hydroxylation as the major pathway of HT-2. 

In phase II reaction samples of HT-2, the 3-glucuronide of HT-2 was generated as described when discussing the observed T-2 metabolites.

#### 2.1.2. Trichothecene Type B

**Figure 4:**
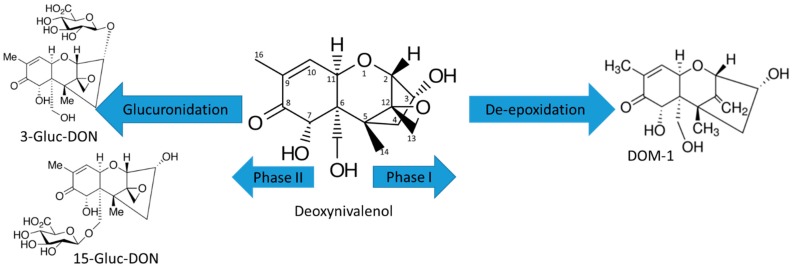
Microsomal biotransformation of DON in phase I and II reactions.

The common phase I pathways of type B trichothecenes are de-acetylation for 3/15-AcDON and FUS-X and de-epoxidation for DON (Figure 4) and NIV. Microsomal biotransformation of DON is summarized in Figure 4 as an example representative for this family. Chromatographic separation and MS^2^ spectra of 3/15-AcDON, FUS-X, DON, NIV and their metabolites are shown in Supplementary Tables S4–S8 and Figures S10–S21. In phase I, all metabolites were generated non-enzymatically—all of these metabolites were observed not only in the phase I sample, but also in controls without NADPH and in heat-inactivated controls. The examples of non-enzymatic reactions included the removal of acetyl group in 3-AcDON converting into DON and de-epoxy-deoxynivalenol DOM-1, 15-AcDON into DON, FUS-X into NIV, DON into DOM-1, and NIV converted into de-epoxy-nivalenol (DNIV), Supplementary Tables S4–S8. 1% of 15-AcDON and 50% of 3-AcDON was converted into DON, whereas 54% of FUS-X was converted into NIV. Only 5% of NIV was converted into DNIV and less than 1% of DON to DNIV. Higher deacetylation rate of 3-AcDON than 15-AcDON had already been demonstrated in literature [44]. In our studies, these metabolites were clearly of non-enzymatic origin; however, other studies have also shown that 3-AcDON can be metabolized to DON (78%) during incubation with human feces [45]. 

During phase II incubations, type B trichothecenes generated 3- and 15-Gluc-DON (1%), shown in Figure 4, Gluc-3-AcDON (11%), and Gluc-15-AcDON (1%) (Supplementary Table S4–S8 and Figures S10–S15, S17–S21), whereas heated samples generated 3- and 15-Gluc-DON (2%), 41% of Gluc-3-AcDON and 2% of Gluc-15-AcDON. However, to observe the glucuronidation of NIV (<1%) and FUS-X (<1%), it was necessary to increase the mycotoxin concentration x10 and incubation time (20 h), and they were only observed in heated samples (Supplementary Figures S18 and S20). The identifications of glucuronides were based on the product ion spectra of [M-H]^−^ for Gluc-FUS-X (529.1561, 0.32 ppm), Gluc-NIV (487.1457, 0.23 ppm), 3- and 15-Gluc-DON (471.1508, 0 ppm), and Gluc-3-AcDON (513.1614, 0.19 ppm) and [M+Na]^+^ for Gluc-15-AcDON (537.1575, 0.74 ppm), Supplementary Tables S4–S8. It is interesting to note that all glucuronides in ESI(-) generated only [M-H]^−^ and not [M+HAc-H]^−^ as their parent mycotoxins. Some mycotoxins, like DON (2 forms), FUS-X (2 forms), and NIV (3 forms), could have more than one glucuronic form based on their structures. In our experiment, we possibly observed two glucuronides of DON based on two distinctive product mass spectra, 3 and 15-Gluc-DON. However, the peaks were not fully resolved and MS^2^ spectra could be a mixture of the two, Supplementary Figures S14 and S17. According to the literature, the first peak can be assigned as 3-Gluc-DON and the second as 15-Gluc-DON [19]. MS^2^ spectrum of the first peak has intense fragment of 441.1 m/z that can happen due to the loss of CH_2_O at position 15 when it is not glucuronidated, Supplementary Figure S17. The partial chromatographic separation of 3-Gluc-DON and 15-Gluc-DON shows that the predominant form is 15-Gluc-DON. FUS-X glucuronide showed only one chromatographic peak as shown in Supplementary Figure S18. Previously, FUS-X glucuronides were not reported either in animal nor human samples (Supplementary Figure S19). NIV glucuronides showed two not fully resolved peaks, assuming that there are at least two glucuronic forms present, Supplementary Figure S20. Only one MS^2^ spectrum was obtained for the second peak, Supplementary Figure S21b. However, previous studies of nivalenol metabolism in rats exhibited only one 3-glucuronide-NIV and DNIV [46]. De-epoxidation of DON also was observed in both human and animals [17,47,48]. In contrast to rat metabolism studies, NIV incubation with human feces showed no de-epoxydated metabolites [45]. To the best of our knowledge, NIV glucuronides have not been previously observed in human samples, possibly due to the low extent of glucuronidation and/or poor limits of detection for the polar nivalenol and its metabolites using most LC-MS methods. The human exposure studies to DON revealed that the predominant species were 15-Gluc-DON (49%), then free DON (27%), and 3-Gluc-DON (14%) in urine and proposed to use them as biomarkers of DON exposure [17]. Despite trichothecene type B mycotoxins, including DON, being extensively studied, we found new metabolites, showing the importance of these detailed incubation studies and the need to build more systematic libraries of mycotoxin metabolites.

### 2.2. Aflatoxins

**Figure 5:**
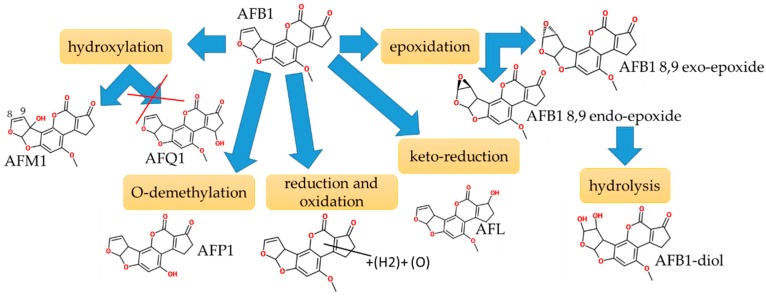
Microsomal biotransformations of aflatoxin B1 in phase I reactions.

AFB1 microsomal biotransformations included the following three types of reactions: oxidative (hydroxylation, epoxidation), reductive (keto-reduction), and hydrolytic (hydrolysis) in phase I, as summarized in Figure 5 and Supplementary Table S9. AFB1 generated various metabolites, including aflatoxin M1 (AFM1, 3%), AFB1 8,9 endo/exo-epoxide (AFBO, <1%), aflatoxin B1 di-hydrodiol (AFB1-diol, <1%), and minor metabolites, AFP1 (<1%), ((H2)+(O))-AFB1 (<1%), and AFL (<1%). The chromatographic separation of AFB1 and its metabolites is shown in Supplementary Figure S22. All metabolites were identified based on their MS1 and comparison of their MS^2^ spectra to the literature data as described in the Supplementary Table S9. Two hydroxy-metabolites at m/z 329.0651 (1.8 ppm) showed shift of 16.0 Da versus [M+H]^+^ ion of AFB1 at 313.0707 (0 ppm), thus confirming the presence of additional oxygen in those compounds, Supplementary Table S9 and Figure S23a,b. The first peak at RT of 5.03 min was identified as AFBO based on the fact of in-source AFB1-diol formation, Supplementary Figures S22 and S23e. AFBO was previously described as a non-stable compound that reacts with water to form AFB1-diol [49,50]. The second peak at RT 6.32 min was identified as hydroxy-metabolite, AFM1. The identification of this hydroxy metabolite was performed by comparing its product ion mass spectra (Supplementary Figure S23b) to the published one [51]. The main distinctive fragment ion of AFM1 is 273.0757 m/z, which can be present only in AFM1 and not in its isomer aflatoxin Q1 (AFQ1) based on the previously published work by Walton et al. [52]. Also, fragment ions of 273.1 Da and 259.0 Da observed in our product ion spectra were chosen as quantifier and qualifier ion for AFM1 in other published papers [51,53,54]. Finally, MS^2^ of AFM1 is similar to product mass spectra obtained by Everley et al. [51]. [M+H]^+^ ion at m/z 347.0760 (0.23 ppm) was 34.0 mass units greater than AFB1, Supplementary Table S9 and Figure S23c,d. This difference indicated the presence of two hydroxyl groups, whereas the presence of two chromatographic peaks indicates the presence of two isomers as shown in Supplementary Figure S22. Their MS^2^ spectra exhibited the intense water loss fragment, 329.0650 (3.3 ppm) and 329.0653 (2.1 ppm) for the first and the second peaks, which confirms the presence of hydroxyl groups, Supplementary Figure S23c,d. Additionally, both peaks showed the loss of two water molecules that yielded fragments, 311.0545 (3.5 ppm) and 311.0549 (2.3 ppm), Supplementary Figure S23c,d. The first peak can be identified as AFB1-diol with hydroxyl groups at positions 8 and 9. Its product mass spectra fragments, 283.0597 (38%) and 329.0650 (100%), have similar intensity as shown by Walton et al., namely, 329.1 (100%) and 283.0 (32%) [52]. The identification of O-demethylated products with theoretical mass of [M+H]^+^ ion at 299.0550 m/z resulted in two chromatographic peaks at 7.05 min (299.0549, 0.33 ppm) and 7.31 min (299.0549, 0.33 ppm), Supplementary Table S9 and Figure S22. Both peaks had similar product ion mass spectra, Supplementary Figure S24. Based on the comparison of fragment ions 271.0602 Da and 299.0554 Da (Supplementary Figure S24) observed in their product ion mass spectra to the literature data, it was possible to determine these peaks as aflatoxin P1 (AFP1) and its isomer [52]. Two metabolites of keto-reduction pathway with measured m/z 337.0682 (0 ppm) of [M+Na]^+^ ion at 7.66 min and 8.60 min were putatively identified as an isomer of aflatoxicol and aflatoxicol (AFL), respectively, since they were found at trace level, Supplementary Table S9 and Figure S22. The conversion of AFB1 to AFL was previously confirmed using in vitro studies of placental human microsomal proteins [55]. One more type of reduction reaction with the further oxidation resulted in metabolites (+(H2)+(O)-AFB1) with m/z 331.0812 (0 ppm) of [M+H]^+^ ion at and RT at 5.45 min, Supplementary Table S9 and Figures S22 and S23f. This metabolite definitely has AFB1 origin, since its product mass spectrum has the same fragments, 285.0757 m/z and 313.0705 m/z as AFB1 (Supplementary Figure S23). Also, the loss of H_2_O (18.0106, 0 ppm) in product ion mass spectra confirmed the OH group in this molecule. Dohnal et al. reviewed aflatoxin metabolism and concluded that besides the interspecies differences there were also regional, inter-individual differences [56]. The main urinary metabolite of AFB1 was AFM1, which was observed in Brazilian volunteers [57]. Also, AFM1 was found in Italian adult urine samples [58] and Italian children urine and serum samples [24]. However, AFQ1 was found as the most predominant form of aflatoxins in Chinese urinary and fecal samples [59]. Also, previously it was shown that different enzymes are responsible for the conversion of AFB1 to AFQ1 and AFM1 [60,61].

The remaining aflatoxins, AFG1, AFB2, AFG2 were unstable during experiment and produced non-enzymatic hydroxyl metabolites. AFG1, AFB2, AFG2 and their metabolites are summarized in Supplementary Tables S10–S12. The stability of aflatoxins in plasma at room temperature was evaluated and it was shown that AFG1 and AFG2 were not stable in plasma for more than 3 h [29]. Another stability study demonstrated the dependence of aflatoxin stability on the temperature and the composition of the solvent [62]. One hydroxy metabolite with enzymatic origin was observed corresponding to hydroxy-metabolite of AFG1, [M+H]^+^ ion at m/z 345.0604 (0.3 ppm), Supplementary Table S11. MS^2^ spectra of AFG1 and its hydroxyl metabolite are shown in Supplementary Figure S25a,b. This AFG1 hydroxy metabolite can be putatively identified as aflatoxin GM1 (AFGM1) metabolite [63]; however, this metabolite, to our knowledge, had not been previously found in human samples. Studies of the prevalence of different aflatoxins in Egyptian infant blood and urine samples performed by Hatem et al. did not confirm its presence [64]. To our knowledge, there were no in vitro metabolism studies performed for AFG1 or AFG2. In our experiment, four non-enzymatic hydroxy metabolites of AFG2 (Supplementary Table S12) were observed with 347.0761 m/z, at least two of them could be aflatoxin GM2 (AFGM2) and aflatoxin G2A (AFG2A) as mentioned in the previous review paper [61]. Product mass spectrum of AFG2 is shown in Supplementary Figure S26. AFB2 was converted non-enzymatically to three hydroxy metabolites with 331.0813 m/z (Supplementary Table S10). Product mass spectra of AFB2 and its hydroxyl metabolites are shown in Supplementary Figure S27a–c. Putatively, they can be identified as previously mentioned aflatoxin M2 (AFM2), aflatoxin Q2 (AFQ2), and aflatoxin B2A (AFB2A) [61,65]. Roebuck et al. performed in vitro metabolism studies of AFB2 which showed the presence of trace levels of AFQ2, aflatoxin P2 (AFP2) and either AFM1 or AFM2 in human samples [66]. In phase II, no glucuronides for any aflatoxins were generated.

### 2.3. Group of Zearalenone

**Figure 6:**
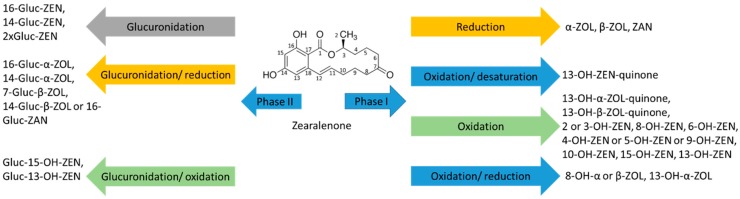
Microsomal biotransformation of ZEN in phase I and II reactions.

**Figure 7:**
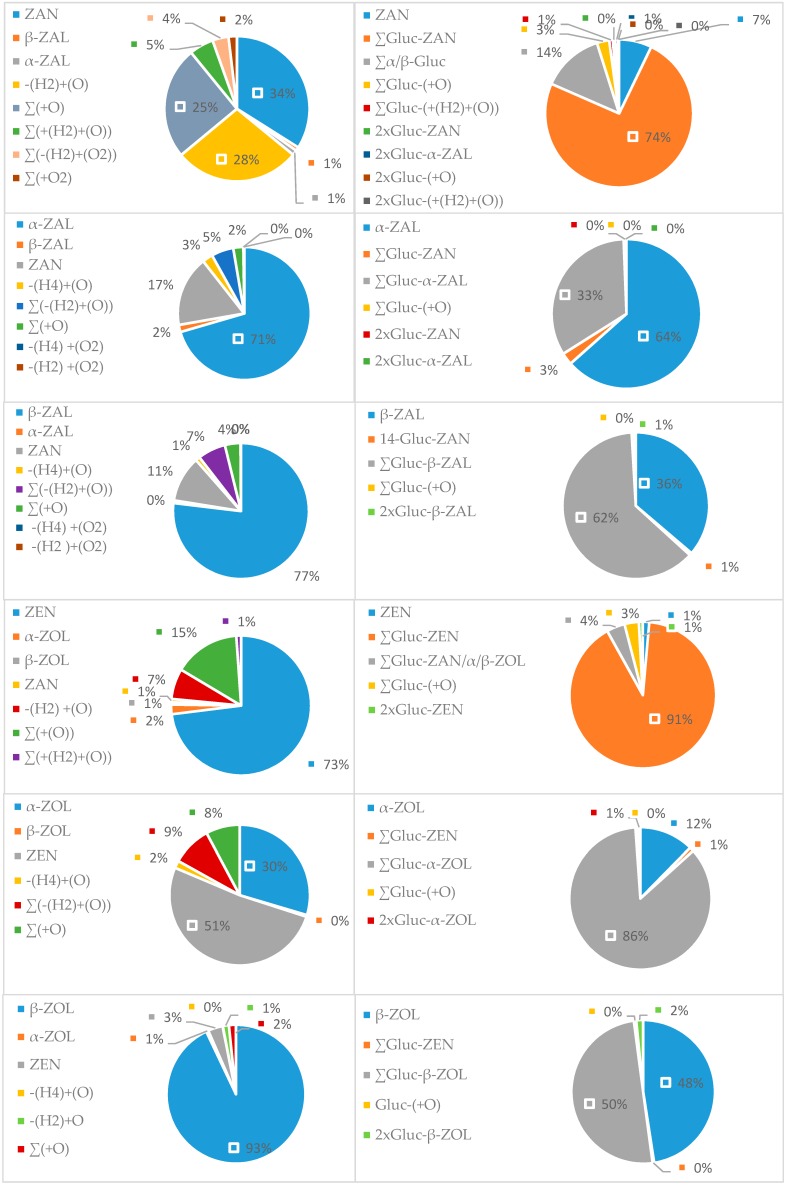
Summary of phase I and phase II metabolism of zearalenone group.

Microsomal biotransformation of ZEN is summarized in Figure 6 as an example representative for this family. The group of zearalenone, ZEN, α-ZOL, β-ZOL, ZAN, α-ZAL, and β-ZAL, was metabolized most extensively out of all chosen mycotoxin groups, resulting in total of 133 metabolites (Figure 7, Supplementary Tables S13–S18). The most predominant phase I metabolic pathway for this class of mycotoxins is oxidation. There were seven types of oxidation reactions, desaturation with oxidation (-(H4) +(O)), desaturation with oxidation (-(H2) +(O)), oxidation (+(O)), reduction with oxidation (+(H2)+ (O)), oxidation (+(O2)), and desaturation with oxidation (-(H2) +(O2) and (-(H4) +(O2)). Among these oxidation (+(O)) reactions resulted in the formation of the highest number of metabolites for ZEN, 9 metabolites, α-ZAL (4), β-ZAL (8), α-ZOL (8), β-ZOL (7), except ZAN for which the reduction with oxidation (+(H2)+ (O)) resulted in the highest number of metabolites (8), as shown in Table 1. Also, the total pattern number of oxidized metabolites of ZEN and its two metabolites, α-ZOL and β-ZOL differed from ZAN and its two metabolites, α-ZAL and β-ZAL. ZAN metabolized the most extensively and resulted in 22 metabolites, but ZEN had only 12 metabolites. α-ZOL (15) had more oxidized metabolites than β-ZOL (8), but α-ZAL (10) had less than β-ZAL (15). According to the percentage of metabolized parent toxin in phase I reactions, ZEN (27%), ZAN (66%), α-ZAL (29%), β-ZAL (23%), α-ZOL (70%), and β-ZOL (7%), this metabolic pathway is not predominant, except for ZEN and α-ZOL. The metabolism of ZEN has already been investigated by Yang et al. [31], and they reported a variety of ZEN oxidized metabolites. Also, ZEN, α-ZAL and ZAN oxidized metabolites were reported in other studies [67,68], but there were no metabolism studies performed for α-ZOL, β-ZOL and β-ZAL. 

Based on the present results, the glucuronidation pathway is the predominant metabolic pathway for almost all the group of zearalenones. 93% of ZAN, 99% of ZEN, 64% of β-ZAL, 51% of β-ZOL, and 88% of α-ZOL were converted into glucoronides, except α-ZAL where conversion was 36% (Figure 7). The identifications of both phase I and II reaction products were based on the comparison MS^2^ spectra to literature data and/or analysis of MS^2^ spectra. Phase II reactions resulted in various glucuronide forms of parent toxin, its metabolites of phase I reactions, and double glucuronide forms (denoted as 2 × Gluc). The numbers of observed glucuronide forms for each mycotoxin from zearalenone group are summarized in Table 2. Common glucuronide forms of parent toxins were glucuronides at position C-14 or C-16 for ZEN and ZAN and at additional position C-7 for α-ZOL, β-ZOL, α-ZAL and β-ZAL. These glucuronides were previously generated by Stevenson et al. [35], and their studies are in accordance with ours. Among the most predominant glucuronides were glucuronides of parent toxins at position C-14, ZEN (86%), ZAN (73%), α-ZOL (72%), β-ZOL (27%), α-ZAL (23%) and β-ZAL (45%). The sum of parent glucuronides of group of zearalenone composed 91% for ZEN, 74% for ZAN, 86% for α-ZOL, 49% for β-ZOL, 33% for α-ZAL and 62% β-ZAL as shown in Figure 7. The glucuronides of oxidized metabolites and double glucuronides were only minor products, 19% for ZAN, 3% for α-ZAL, 2% for β-ZAL, 8% for ZEN, 2% for α-ZOL, 2% for β-ZOL. Yang et al. have already reported ZEN glucuronides of oxidized metabolites and di-glucuronide forms [69]. Overall, ZAN was the most metabolized toxin in phase II and resulted in 24 glucuronides. β-ZOL and β-ZAL were least metabolized toxins and each generated only 7 glucoronic forms. Comparing phase II reaction samples to heated controls, it was noticed that the glucururonidation process was more efficient in heated samples (45 °C) vs. phase II reaction samples, except ZAN. 

## 4. Conclusions

In conclusion, the newly generated LC-MS library containing 188 metabolites represents the most comprehensive resource of mycotoxin metabolites that can be analyzed using a single LC-MS method. The in vitro microsomal incubation workflow used in this work was able to successfully generate metabolites from hydrolysis, oxidation, de-epoxidation, epoxidation, demethylation, reduction and glucuronidation pathways as summarized in Table 3. The excellent limits-of-detection and isomer separation capability of our LC-MS method allowed us to characterize for the first time 100 metabolites that had not been previously reported in the literature, to the best of our knowledge. Among the known phase I and phase II metabolites of 17 mycotoxins that were the focus of this study, only four metabolites—aflatoxin Q1 (AFQ1), aflatoxin P2 (AFP2), Gluc-4-HT-2, Gluc-3-4-de-acetyl-neosolaniol—could not be generated using our microsomal incubation workflow. The remaining 88 known metabolites were successfully generated, thus showing the power of our workflow and high-confidence identification capability. Table 4 summarizes the main subclasses of the newly characterized metabolites in this study. To ensure the high confidence of our library identifications we used three key strategies: (i) incubation with one mycotoxin at a time to properly assign the origin of metabolites to a given parent mycotoxin, (ii) extensive controls to eliminate endogenous biomolecules present in microsomes, impurities in standards and metabolites that could be generated non-enzymatically, and (iii) MS/MS comparison to the published literature spectra when available and to the parent compounds since the generated metabolites share many of the same structural features as the parent compounds. In the absence of authentic standards for all of these metabolites, our identifications are putative. In future, this new LC-MS library will be used during biomonitoring studies to characterize which of these metabolites may be observed in various biological samples in vivo and to provide semi-quantitative information on their concentrations using parent calibration curves. This relevant subset of metabolites then can be synthesized for further confirmation of identity and full quantification. Additionally, the clarification of some metabolite structures that remain ambiguous in our library (e.g., exact position of hydroxyl groups in several ZEN metabolites) can be improved in future work by the application of isotopically labeled standards, as was previously demonstrated in the literature [68,70], or through synthesis of authentic standards.

## 5. Materials and Methods 

### 5.1. Chemicals

Water ((H_2_O, LC-MS grade), methanol ((MeOH), LC-MS grade), acetonitrile ((MeCN), LC-MS grade), and acetic acid ((AA, LC-MS grade) were purchased from Fisher Scientific (Ottawa, Ontario, Canada). Sodium chloride ((NaCl), meets specifications of American Chemical Society grade (ACS), ≥99.0%), sodium phosphate dibasic ((Na_2_HPO_4_), ACS, ≥99.0%), potassium phosphate monobasic ((KH2PO_4_), ACS, ≥99.0%), and magnesium chloride ((MgCl_2_),anhydrous, ≥98%), β-nicotinamide adenine dinucleotide 2′-phosphate reduced tetrasodium salt hydrate ((NADPH), ≥97%), uridine 5′-diphosphoglucuronic acid trisodium salt ((UDPGA), 98–100%), alamethicin from *Trichoderma viride* (≥98%, HPLC grade), and human microsomes from liver (pooled, CMV-negative, 20 mg/mL) were purchased from Sigma-Aldrich Canada (Oakville, Ontario, Canada). Potassium chloride ((KCl), reagent grade, 99.0%) was purchased from BioShop Canada (Burlington, Ontario, Canada). 

### 5.2. Mycotoxin Standards

All mycotoxins were purchased from Sigma-Aldrich Canada, unless otherwise indicated. AFG1, T-2, HT-2, α-ZAL, β-ZAL were purchased from Toronto Research Chemicals Inc. (Toronto, ON, Canada). Zearalenone (ZEN) was purchased from Cayman Chemicals (Ann Arbor, MI, USA). Individual standard stock solutions of all mycotoxins at 1 mg/ml concentration were prepared in methanol and kept at −80 °C. 

### 5.3. Experimental Design and Microsomal Incubations

The purpose of this work was to generate phase I and phase II (glucuronidation) metabolites of 17 mycotoxins using standard in vitro microsomal incubation protocol. Each toxin was incubated individually with microsomes in the presence of NADPH for phase I reactions. For phase II glucuronidation reactions, UDPGA, alamethicin and MgCl_2_ were also added. In all cases, the following controls were used in order to confirm product formation during enzymatic reaction: (i) microsomal incubation without toxin added, (ii) microsomal incubation without co-factors added (iii), microsomal incubation without NADPH, but containing UDPGA, alamethicin and MgCl_2_, (iv) incubation with heated microsomes, and (v) standard solution of each toxin dissolved in PBS buffer. This experimental design is summarized in Figure 1. 

100 mM PBS buffer (pH 7.4), 20 mM NADPH dissolved in 100 mM phosphate buffer, 100 mM UDPGA in water, 5 mg/mL alamethicin in methanol, 100 mM MgCl_2_ in water and 200 µg/mL standard solution of each mycotoxin in acetonitrile were prepared before the start of microsomal incubations. Microsomes were thawed on ice. In an Eppendorf tube, 182 µL of PBS buffer, 2 µL of NADPH and 5 µL of microsomes were transferred for phase I reactions. For phase II reactions, all of the reagents for phase I reactions plus alamethicin, 10 µl of UDPGA and MgCl_2_ were transferred. Microsomes were then pre-incubated for 5 min, followed by the addition of mycotoxin (final concentration of 1 µg/mL) and then the remaining amount of NADPH (10 µL). All samples were incubated for 1 h at 37 °C, reactions were stopped by adding 200 µL of acetonitrile. Detailed description of test samples and controls is shown in Supplementary Table S1. 

### 5.4. LC-HRMS Analysis

All LC-MS measurements were performed according to the validated multi-mycotoxin method for 17 parent mycotoxins [29]. Briefly, the method combined HPLC 1100 (Agilent Technologies, Santa Clara, CA, USA) and reversed-phase chromatographic separation on pentafluorophenyl stationary phase and gradient elution using water and methanol containing 0.1% AA (v/v) for ESI(+), and 0.02% for ESI(-) [29]. The flow rate of 0.3 mL/min, the column temperature of 30°C, and 10 µL injection volume were used for all analyses. MS analysis was performed on LTQ Orbitrap Velos at 60,000 resolving power using the mass range of 200–700 m/z. In addition, MS^n^ analysis with data-dependent acquisition (DDA) mode was used for the identification and elucidation of metabolite structures. In DDA mode, the three most intense ions from the full MS scan were selected for MS^2^ fragmentation. MS^2^ analysis used collision-induced dissociation (CID) and signal threshold: 5,000; normalized collision energy: 35; isolation width: 2 Da; activation time: 30 ms. MS^3^ used targeted parent and product mass lists to trigger MS^3^ for the selected ions of interest. MS^3^ was performed with CID as activation type; minimal signal threshold: 5000; isolation width: 2 Da; activation time: 30 ms; normalized collision energy: 45. For AFB1 and its metabolites, MS^2^ analysis used higher energy collisional dissociation (HCD) with signal threshold: 5000; normalized collision energy: 35; isolation width: 2 Da; activation time: 0.1 ms, lock mass was used for ESI(−) and ESI(+). 

Data was processed using Compound Discoverer 2.1 (ThermoFisher Scientific). Raw data files were uploaded to Compound Discoverer and analyzed using generic metabolism workflow. General settings in the workflow were mass tolerance, 5 ppm; signal threshold, 3; minimum peak intensity 10000. Parameters used to generate expected compounds were parent toxin structure, metabolic transformations for phase I and II reactions, and preferred ions.

## Figures and Tables

**Table 1:** Summary of Phase I oxidation metabolites observed for zearalenone group

Mycotoxin	Oxidation Reactions and Number of Metabolites, (*n*)							∑ n
	Desaturation, oxidation, −(H4) +(O)	Desaturation, oxidation, −(H2) +(O)	Oxidation +(O)	Reduction, oxidation, +(H2) +(O)	Oxidation, +(O2)	Desaturation oxidation, −(H2) +(O2)	Oxidation, −(H4) +(O2)	
ZEN	0	1	9(75%)	2	0	0	0	12
α-ZOL	1	6	8(53%)	0	0	0	0	15
β-ZOL	1	1	7(88%)	0	0	0	0	8
ZAN	0	1	5(23%)	8(36%)	6(27%)	2	0	22
α-ZAL	1	3	4(40%)	0	0	1	1	10
β-ZAL	1	4	8(53%)	0	0	1	1	15

**Table 2:** Number of glucuronides observed in zearalenone group.

Mycotoxin	Total Number of Glucuronides
ZEN	10
α-ZOL	10
β-ZOL	7
ZAN	24
α-ZAL	10
β-ZAL	7

**Table 3:** Summary of metabolic pathways of 17 mycotoxins.

Mycotoxins	Hydrolysis	Oxidation	De-Epoxidation	Epoxidation	Demethylation	Reduction	Glucuronidation
T-2	✓	✓					✓
HT-2	✓	✓					✓
3-AcDON	✓						✓
15-AcDON	✓						✓
FUS-X	✓						✓
DON			✓				✓
NIV			✓				✓
AFB1	✓	✓		✓	✓	✓	
AFB2		✓					
AFG1		✓					
AFG2		✓					
ZEN		✓					✓
α-ZOL		✓					✓
β-ZOL		✓					✓
ZAN		✓					✓
α-ZAL		✓					✓
β-ZAL		✓					✓

**Table 4:** Comparison of literature expected metabolites and generated LC-MS library metabolites.

Mycotoxin	Expected Metabolites	Missing Metabolites	LC-MS Library	New Metabolites
T-2	Phase I metabolites: HT-2, 15-deacetyl-T-2 (15-de-Ac-T-2), 3′-OH-T-2, neosolaniol (NEO), T-2 triol, 3′-OH-HT-2, T-2 triol, Glucuronides: Gluc-3-T-2	NO	Phase I metabolites: HT-2, 15-de-Ac-T-2, 3′-OH-T-2 and its two isomers, NEO or T-2 triol, 3′-OH-HT-2, Glucuronides: Gluc-3-T-2	Two isomers of 3′-OH-T-2, 4 isomers of 3′-OH-HT-2
HT-2	Phase I metabolites: 4-de-Ac-NEO, 3′-OH-HT-2, 4′-OH-HT-2 7-OH-HT-2 and its isomer, 10-OH-HT-2, Glucuronides: Gluc-3-HT-2, Gluc-4-HT-2, Gluc-3-4-de-Ac-NEO	Gluc-4-HT-2, Gluc-3-4-de-Ac-NEO	Phase I metabolites: 4-de-Ac-NEO and its isomer, 3′-OH-HT-2, 4′-OH-HT-2 and its isomer Three OH-T-2 metabolites at 7 or 10 or 16-OH-HT-2 Two unknown metabolites Glucuronides: Gluc-3-HT-2	4-de-Ac-NEO isomer
3-AcDON	Phase I metabolites: DON Glucuronides: Gluc-3-AcDON	NO	Phase I metabolites: DON Glucuronides: Gluc-3-AcDON	NO
15-AcDON	Phase I metabolites: DON Glucuronides: Gluc-15-AcDON	NO	Phase I metabolites: DON Glucuronides: Gluc-15-AcDON	NO
FUS-X	Phase I metabolites: NIV	NO	Phase I metabolites: NIV Glucuronides: Gluc-FUS-X	Gluc-FUS-X
DON	Phase I metabolites: De-epoxy-DON (DOM-1) Glucuronides: 15-Gluc-DON, 3-Gluc-DON	NO	Phase I metabolites: NIV, DOM-1 and its two isomers, Glucuronides: 15-Gluc-DON, 3-Gluc-DON	NIV, isomers of DOM-1
NIV	Phase I metabolites: De-epoxy-NIV (DENIV), Glucuronides: Gluc-3-NIV	NO	Phase I metabolites: DENIV and its two isomer Glucuronides: Two Gluc-NIVs	Gluc-NIV
AFB1	Phase I metabolites: AFM1, AFQ1, AFBO, AFP1, AFL, AFB1-diol Glucuronides: NO	AFQ1	Phase I metabolites: AFM1, AFBO, AFP1 and its isomer, AFL and its isomer, AFB1-diol and its isomer, ((H2)+(O)-AFB1 Glucuronides: NO	((H2)+(O)-AFB1
AFB2	Phase I metabolites: AFM2, AFQ2, AB2A, AFP2 Glucuronides: NO	AFP2	Phase I metabolites: AFM2, AFQ2 and AFB2A Glucuronides: NO	NO
AFG1	Phase I metabolites: AFGM1 Glucuronides: NO	NO	Phase I metabolites: AFGM1 Glucuronides: NO	NO
AFG2	Phase I metabolites: AFGM2, AFG2A Glucuronides: NO	NO	Phase I metabolites: AFGM2, AFG2A Glucuronides: NO	NO
ZEN	Phase I metabolites: (-(H2) +(O))-ZEN, (+(O))-ZEN, (+(H2)+ (O))-ZEN Glucuronides: Gluc-16-ZEN, Gluc-14-ZEN, Gluc-(+O)-ZEN, 2xGluc-ZEN	NO	Phase I metabolites: (-(H2) +(O))-ZEN, (+(O))-ZEN, (+(H2)+ (O))-ZEN Glucuronides: Gluc-16-ZEN, Gluc-14-ZEN, Gluc-(+O)-ZEN, 2xGluc-ZEN	NO
α-ZOL	Phase I metabolites: (-(H4)+(O))- α-ZOL (-(H2)+(O))- α-ZOL (+O)- α-ZOL Glucuronides: Gluc-16- α-ZOL, Gluc-14- α-ZOL, Gluc-7- α-ZOL	NO	Phase I metabolites: (-(H4)+(O))- α-ZOL (-(H2)+(O))- α-ZOL (+O)- α-ZOL Glucuronides: Gluc-16- α-ZOL, Gluc-14- α-ZOL, Gluc-7- α-ZOL, Gluc-(+O)- α-ZOL, 2 × Gluc- α-ZOL	Gluc-(+O)- α-ZOL, (2 × Gluc)- α-ZOL
β-ZOL	Phase I metabolites: NO Glucuronides: Gluc-16- β-ZOL, Gluc-14- β-ZOL, Gluc-7- β-ZOL,	NO	Phase I metabolites: (-(H4)+(O))- β-ZOL (-(H2)+(O))- β-ZOL (+O)- β-ZOL Glucuronides: Gluc-16- β-ZOL, Gluc-14- β-ZOL, Gluc-7- β-ZOL, Gluc-(+O)- β-ZOL, 2 × Gluc- β-ZOL	(-(H4)+(O))- β-ZOL (-(H2)+(O))- β-ZOL (+O)- β-ZOL Gluc-(+O)- β-ZOL, (2 × Gluc)- β-ZOL
ZAN	Phase I metabolites: NO Glucuronides: Gluc-16- ZAN, Gluc-14-ZAN	NO	Phase I metabolites: (-(H2) +(O))-ZAN, (+(O))-ZAN, (+(H2)+ (O))-ZAN, (+(O2))-ZAN, (-(H2) +(O2))-ZAN Glucuronides: Gluc-16-ZAN, Gluc-14-ZAN, Gluc-(+O)- ZAN, 2xGluc- ZAN, Gluc-(+(H2)+(O))-ZAN, 2 × Gluc-(+O)-ZAN, 2 × Gluc-(+(H2)+(O))-ZAN	Gluc-(+O)- ZAN, (2 × Gluc)- ZAN, Gluc-(+(H2)+(O))-ZAN, (2 × Gluc)-(+O)-ZAN, (2 × Gluc)-(+(H2)+(O))-ZAN
α-ZAL	Phase I metabolites: (-(H4)+(O))- α-ZAL (-(H2)+(O))- α-ZAL (+O)- α-ZAL (-(H4) +(O2))- α-ZAL (-(H2 )+(O2))- α-ZAL Glucuronides: Gluc-16- α-ZAL, Gluc-14- α-ZAL, Gluc-7- α-ZAL,	NO	Phase I metabolites: (-(H4)+(O))- α-ZAL (-(H2)+(O))- α-ZAL (+O)- α-ZAL (-(H4) +(O2))- α-ZAL (-(H2 )+(O2))- α-ZAL Glucuronides: Gluc-16- α-ZAL, Gluc-14- α-ZAL, Gluc-7- α-ZAL, Gluc-(+O)- α-ZAL 2 × Gluc-α-ZAL	Gluc-(+O)- α-ZAL (2 × Gluc)-α-ZAL
β-ZAL	Phase I metabolites: NO Glucuronides: Gluc-16-β-ZAL, Gluc-14-β-ZAL, Gluc-7-β-ZAL	NO	Phase I metabolites: (-(H4)+(O))- β-ZAL (-(H2)+(O))- β-ZAL (+O)- β-ZAL (-(H4) +(O2))- β-ZAL (-(H2 )+(O2))- β-ZAL Glucuronides: Gluc-16-β-ZAL, Gluc-14-β-ZAL, Gluc-7-β-ZAL, Gluc-(+O)- β-ZAL, 2xGluc-β-ZAL	(−(H4)+(O))- β-ZAL (−(H2)+(O))- β-ZAL (+O)- β-ZAL (−(H4) +(O2))- β-ZAL (−(H2 )+(O2))- β-ZALGluc-(+O)- β-ZAL, (2xGluc)-β-ZAL

## Supplementary Materials

The following are available online at https://www.mdpi.com/2072-6651/11/8/433/s1, Table S1: Microsomal incubation protocol for phase I and II reactions, Table S2: T-2 and its metabolites, detected in ESI(+), as CID product ion spectra of [M+Na]^+^, Table S3: HT-2 and its metabolites, detected in ESI(+), as [M+Na]^+^ ions, Table S4: Metabolites of 3-AcDON generated in phase I and phase II, detected in ESI(−), as [M+CH_3_COO-H]^−^ ions, except Gluc-3AcDON which was detected as [M-H]^−^ ion, Table S5: Metabolites of 15-AcDON generated in phase I and phase II, detected in ESI(+), as [M+Na]^+^ ions of 15-AcDON and Gluc-15-AcDON, except DON which was detected as [M+H]^+^ ion, Table S6: Metabolites of DON generated in phase I and phase II, detected in ESI(−), as [M+CH_3_COO-H]^−^ ions, except Gluc-DON which was detected as [M-H]^−^ ion, Table S7: Metabolites of FUS-X generated in phase I and phase II, detected in ESI(−), as [M+CH_3_COO-H]^−^ ions, except Gluc-FUS-X which was detected as [M-H]^−^ ion, Table S8: Metabolites of NIV generated in phase I and phase II, detected in ESI(−), as [M+CH_3_COO-H]^−^ ions, except Gluc-NIV which was detected as [M-H]^−^ ion, Table S9: AFB1 and its metabolites of phase I reactions, detected in ESI(+), as [M+H]^+^ ions and * [M+Na]^+^ ions for AFL, Table S10: AFB2 and its metabolites of phase I reactions, detected in ESI(+), as [M+H]^+^ ions, Table S11: AFG1 and its metabolite, OH-AFG1, detected in ESI(+), as [M+H]^+^ ions, Table S12: AFG2 and its metabolites of phase I reactions, detected in ESI(+), as [M+H]^+^ ions, Table S13: Metabolites of ZEN generated in phase I and phase II, detected in ESI(−), as [M-H]^−^ ions, Table S14: Metabolites of α-ZOL generated in phase I and phase II, detected in ESI(−), as [M-H]^−^ ions, Table S15: Metabolites of β-ZOL generated in phase I and phase II, detected in ESI(−), as [M-H]^−^ ions, Table S16: Metabolites of ZAN generated in phase I and phase II, detected in ESI(−), as [M-H]^−^ ions, Table S17: Metabolites of α-ZAL generated in phase I and phase II, detected in ESI(−), as [M-H]^−^ ions, Table S18: Metabolites of β-ZAL generated in phase I and phase II, detected in ESI(−), as [M-H]^−^ ions, Figure S1: Extracted ion chromatograms of T-2 and its metabolites (505.2044 m/z, peak 1-505 to peak 3-505, 463.1939 m/z, peak 1-463 to peak 5-463, 405.1884 m/z, peak 1-405, 447.1989 m/z, peak 1-447 and peak 2-448) in ESI(+), detected as [M+Na]^+^ ions, Figure S2: Zooming into extracted ion chromatograms of T-2 (505.2044 m/z) and HT-2 (463.1938 m/z) hydroxy metabolites in ESI(+), detected [M+Na]^+^ ions. Panel (a) shows T-2 hydroxyl metabolites, panel (b) shows HT-2 hydroxyl metabolites, Figure S3: Product ion spectra of T-2 hydroxy metabolites at 505.2044 m/z, peak 1-505 (a), peak 2-505 (b), peak 3-505 (c) and at 489.2095, T-2, detected in ESI(+), as [M+Na]^+^ ions, Figure S4: Product ion mass spectra of the peak 1-447 (a) which was tentatively identified as 15-deacetyl-T-2 and the peak 2-447 (b) which was identified as HT-2, detected in ESI(+), as [M+Na]^+^ ions, Figure S5: Extracted ion chromatograms of T-2 and HT-2 glucuronides (665.2416 m/z, Gluc-T-2, and 623.2310 m/z, Gluc-HT-2) in ESI(+), detected as [M+Na]^+^ ions, Figure S6: Product ion spectra of HT-2 glucuronide (a) at 623.2310 m/z; T-2 glucuronide (b) at 665.2416 m/z and HT-2 glucuronide (c) at 618.2756 m/z, detected in ESI(+), as [M+Na]+ ions for (a) and (b) and [M+NH4]+ions for (c), Figure S7: Extracted ion chromatograms of HT-2 and its metabolites (463.1939 m/z, peak 1-463 to peak 5-463, 405.1884 m/z, peak 1-405 and peak 2-405, 363.1414 m/z, peak 1-363 and peak 2-363) in ESI(+), detected as [M+Na]^+^ ions, Figure S8: Zooming into extracted ion chromatogram of HT-2 hydroxyl-metabolites (463.1939 m/z, peak 1-463 to 5-463), Figure S9: Product ion mass spectra of HT-2 hydroxy metabolites at 463. 1939 m/z: peak 1-463(a), peak 2-463 (b), peak 3-463 (c), peak 4-463 (d), peak 5-463 (e), peak 6-463 (f), detected in ESI(+), as [M+Na]^+^ ions, Figure S10: Extracted ion chromatograms of 3-AcDON (397.1505 m/z) and its metabolites (355.1399 m/z, DON, 339.1448 m/z, DOM-1, and 513.1613 m/z, Gluc-3-AcDON) in ESI(-), detected as [M+CH_3_COO-H]^−^ ions for all except Gluc-3AcDON ([M-H]^−^), Figure S11: Product ion mass spectra of 3-AcDON at 397.1505 m/z and its glucuronide at 513.1613 m/z, detected in ESI(-), as [M+CH_3_COO-H]^−^ and Gluc-3AcDON ([M-H]^−^ ions, respectively, Figure S12: Extracted ion chromatogram of 15-AcDON (361.1258 m/z) and its metabolites (297.1333 m/z, DON, and 537.1579 m/z, Gluc-15-AcDON) in ESI(+), detected as [M+Na]^+^ ions, Figure S13: Product mass spectra of 15-AcDON (361.1258 m/z) and its glucuronide (537.1579 m/z), detected in ESI(+) as [M+Na]^+^ ions, Figure S14: Extracted ion chromatograms of DON and its metabolites (371.1348 m/z, NIV, 339.1449 m/z, peak 1-339 to peak 4-339, and 471.1508 m/z, Gluc-DON) in ESI(-), detected as [M+CH3COO-H]^−^ ions for all except Gluc-DON ([M-H]^−^), Figure S15: Product mass spectra of DON (355.1399 m/z) and NIV (371.1348 m/z) detected in ESI(-), as [M+CH_3_COO-H]^−^ ions, Figure S16: Product ion mass spectra of de-epoxy-deoxynivalenol at 339.1348 m/z, detected in ESI(-), as [M+CH_3_COO-H]^−^ ions. Peak 1-339 (a) was observed as phase I metabolite of DON and 3-AcDON, peak 2-339 (b) and peak 3-339 (c) were observed as phase I metabolite of DON only, Figure S17: Product mass spectra of DON glucuronides, 471.1508 m/z, detected in ESI(-), as [M-H]^−^ ions, Figure S18: Extracted ion chromatogram of FUS-X (413.1454 m/z) and its metabolites (371.1348 m/z, NIV, and 529.1563 m/z, Gluc-FUS-X) in ESI(-), detected as [M+CH3COO-H]^−^ ions for all except Gluc-FUS-X ([M-H]^−^), Figure S19: Product ion mass spectra of FUS-X at 413.1454 m/z and its glucuronide at 529.1563 m/z, detected in ESI(-), as [M+CH_3_COO-H]^−^ and Gluc-3AcDON ([M-H]^−^ ions, respectively. Figure S20: Extracted ion chromatograms of NIV, de-epoxy-metabolite and its isomers (355.1398 m/z, peak 1-355, peak 2-355, peak 3-355) and its glucuronides (487.1457 m/z) in ESI(-), NIV and de-epoxy-metabolite were detected as [M+CH3COO-H]^−^ ions and glucuronides as [M-H]^−^ ion, Figure S21: Product ion mass spectra of NIV metabolites: de-epoxy-nivalenol, peak 1-355 (a) at 355.1398 m/z and NIV glucuronide (b) at 487.1458 m/z, detected in ESI(-), as [M-H]^−^ and [M+CH_3_COO-H]^−^ ions, respectively., Figure S22: Chromatographic separation of AFB1 metabolites generated in phase I reactions. Extracted ion chromatograms of AFB1 (313.0707 m/z), 337.0682 m/z (peak 1-337 and peak 2-337), 299.0550 m/z (peak 1-299 and peak 2-299), 331.0812 m/z (peak 1-331), 329.0661 m/z (peak 1-329, AFBO, and peak 2-329, AFM1), 347.0761 m/z (peak 1-347 and peak 2-347) detected in ESI(+), Figure S23: Product mass spectra of AFB1 (a); AFM1, peak 2-329 (b); AFB-diol, peak 1 (c); AFB-diol, peak 2 (d); AFB-8,9-endo/exo-epoxide (AFBO), peak 1-329 (e); peak 1-331 (f), detected in ESI(+), as [M+H]^+^ ions, Figure S24: Product ion mass spectra of the peak 1-447 (a) which was tentatively identified as 15-deacetyl-T-2 and the peak 2-447 which was identified as HT-2, detected in ESI(+), as [M+Na]^+^ ions, Figure S25: Product mass spectra of AFG1 (a) at 329.0656 m/z and its hydroxyl metabolite peak 1-345 (b), detected in ESI(+), as [M+H]^+^ ions, Figure S26: Product mass spectra of AFG2 at 331.0813 m/z, detected in ESI(+), as [M+H]^+^ ion:. Figure S27 Product mass spectra of AFB2 (a) at 315.0863 m/z and its hydroxyl metabolites at 331.0813, peak 2-331 (b) and peak 1-331 (c), detected in ESI(+), as [M+H]^+^ ions,
