# Supplementary material for: Characterization of Phase I and Glucuronide Phase II Metabolites of 17 Mycotoxins Using Liquid Chromatography—High-Resolution Mass Spectrometry
Source: Toxins (Basel). 2019 Jul 24;11(8):433. doi: 10.3390/toxins11080433 (PMC6723440; doi:10.3390/toxins11080433)
Supplement: Supplementary file 1 [file toxins-11-00433-s001.pdf]

Supplementary information: Characterization of phase I and glucuronide metabolites of 17 mycotoxins using liquid chromatography – high-resolution mass spectrometry

Irina Slobodchikova, Reajean Sivakumar, Md Samiur Rahman and Dajana Vuckovic

Table S1. Microsomal incubation protocol for phase I and II reactions. *Heated microsomes at T 45 ℃ for 30 min.

Phase I reaction							
Sample type	Mycotoxin volume, µL	Microsome volume, µL	NADPH volume, µL	PBS buffer volume, µL			
Test	1	5	12	182			
Control 1	0	5	12	182			
Control 2	1	5	0	194			
Control 3	1	5 *	12	182			
Control 4	1	0	0	199			
Phase II reaction							
Sample type	Mycotoxin volume, µL	Microsome volume, µL	NADPH volume, µL	UDPGA volume, µL	Alamethecin volume, µL	$MgCl_2$ volume, µL	PBS buffer volume, µL
Test	1	5	12	10	1	10	183
Control 5	0	5	12	10	1	10	184
Control 6	1	5	0	10	1	10	195
Control 7	1	5 *	12	10	1	10	183

Table S2. T-2 and its metabolites, detected in ESI(+), as CID product ion spectra of $[M+Na]^+$, unless otherwise specified fragments with intensity >10% are shown in the table, *fragmentation pattern are shown for $[M+NH_4]^+$ions.

T-2 phase I metabolites								
**Name**	**RT, min**	**Measured m/z**	**Theoretical m/z**	**ppm**	**Fragments, CID**	**Formula**	**Transformation**	**Comments**

T-2	12.23	489.2092	489.2095	0.61	245.2(10), 327.2(37), 387.2(100)	$C_{24}H_{34}O_9$	parent	
Peak 1-447	7.93	447.1988	447.1989	0.22	285.2(21), 345.2(100)	$C_{22}H_{32}O_8$	-(C2 H2 O)	Non enzymatic, 15-deacetyl-T-2
Peak 2-447	8.22	447.1986	447.1989	0.67	285.2(20), 345.2(100)	$C_{22}H_{32}O_8$	-(C2 H2 O)	Non enzymatic, HT-2
Peak 1-505	6.54	505.2042	505.2044	0.40	327.2(27), 387.2(100)	$C_{24}H_{34}O_{10}$	+(O)	*2'-OH-T-2 or 3'-OH-T-2 or 4'-OH-T-2*
Peak 2-505	6.60	505.2041	505.2044	0.59	327.2(22), 387.2(100)	$C_{24}H_{34}O_{10}$	+(O)	*2'-OH-T-2 or 3'-OH-T-2 or 4'-OH-T-2*
Peak 3-505	6.81	505.2043	505.2044	0.20	327.2(10), 387.4(100)	$C_{24}H_{34}O_{10}$	+(O)	*2'-OH-T-2 or 3'-OH-T-2 or 4'-OH-T-2*
Peak 1-463	5.68	463.1936	463.1939	0.65	285.1(16), 345.2(100), 446.0(12)	$C_{22}H_{32}O_9$	-(C2 H2)	3' or 4'-Hydroxy-HT-2
Peak 2-463	5.76	463.1938	463.1939	0.22	345.43(100)	$C_{22}H_{32}O_9$	-(C2 H2)	3' or 4'-Hydroxy-HT-2
Peak 3-463	5.94	463.1938	463.1939	0.22	No MS2	$C_{22}H_{32}O_9$	-(C2 H2)	2'-OH-T-2
Peak 4-463	6.12	463.1937	463.1939	0.43	No MS2	$C_{22}H_{32}O_9$	-(C2 H2)	*Low intensity, 7-OH-HT-2 10-OH-HT-2 16-OH-HT-2*
Peak 5-463	6.21	463.1936	463.1939	0.65	301.16(21), 361.2(100), 381.02(20), 433.99(10), 445.99(26)	$C_{22}H_{32}O_9$	-(C2 H2)	*7-OH-HT-2 or 10-OH-HT-2 or 16-OH-HT-2*
Peak 1-405	6.55	405.1881	405.1884	0.74	No MS2	$C_{19}H_{26}O_8$	-(C5 H8 O)	Low intensity, NEO or T-triol

T-2 phase II metabolites								
Name	RT, min	Measured m/z	Theoretical m/z	ppm	Fragments, CID	Formula	Transformation	Comments
Gluc-T-2	8.88	665.2413	665.2416	0.43	489.23 100	$C_{30}H_{42}O_{15}$	+(C6H8O6)	
Gluc-HT-2	6.91	623.2304	623.2310	0.96	263.17(13) 425.23(6) 442.96(35) 499.12(3) 601.04(100)* and 447.47(100)	$C_{28}H_{40}O_{14}$	+(C4H6O5)	HT-2 3-glucuronide

Table S3. HT-2 and its metabolites, detected in ESI(+), as $[M+Na]^+$ ions. Unless otherwise specified fragments with intensity >10% are shown in the table. * Intensities of fragments are more than 40% shown only; ** fragments with intensity >19% are shown.

HT-2 phase I metabolites								
Name	RT, min	Measured m/z	Theoretical m/z	ppm	Fragments, CID	Formula	Transformation	Comments
HT-2	8.22	447.1986	447.1989		285.2(13), 345.3(100)	$C_{22}H_{32}O_8$	parent	
Peak 1-463	5.67	463.1936	463.1939	0.65	285.2(10), 345.5(100)	$C_{22}H_{32}O_9$	+(O)	3ʹ or 4’-Hydroxy-HT-2
Peak 2-463	5.77	463.1936	463.1939	0.65	285.2(14), 345.2(100)	$C_{22}H_{32}O_9$	+(O)	3ʹ or 4’-Hydroxy-HT-2
Peak 3-463	5.95	463.1936	463.1939	0.65	285.3(19), 345.2(100), 431.2(19), 445.2(79), 446.1(35), 454.4(20), 457.4(25), 463.2(32)**	$C_{22}H_{32}O_9$	+(O)	Low intensity

Peak 4-463	6.11	463.1936	463.1939	0.65	301.1(31), 361.2(100), 445.2(15)	$C_{22}H_{32}O_9$	+(O)	
Peak 5-463	6.21	463.1936	463.1939	0.65	301.2(24), 361.2(100)	$C_{22}H_{32}O_9$	+(O)	
Peak 6-463	8.23	463.1936	463.1939	0.65	301.2(30), 345.3(11), 361.2(100), 403.2(85), 421.2(17), 445.2(17)	$C_{22}H_{32}O_9$	+(O)	Low intensity peak
Peak 1-405	5.44	405.1880	405.1884	0.99	303.2(100), 323.1(17), 325.3 (59), 345.2(19), 360.5(10), 387.2(42), 395.4(14), 396.1(19), 396.8(11)	$C_{20}H_{30}O_7$	-(C2H2O)	Low intensity peak
Peak 2-405	6.55	405.1880	405.1884	0.99	303.2(100), 323.1(36), 345.1(27), 361.2(10), 373.4(19), 387.2(36), 395.6(26), 396.5(23), 404.9(12)	$C_{20}H_{30}O_7$	-(C2H2O)	Low intensity peak
Peak 1-363	3.58	363.1413	363.1414	0.28	303.1(100), 345.2(11)	$C_{17}H_{24}O_7$	-(C5H8O)	4-de-Ac neosolaniol

Peak 2-363	4.87	363.1413	363.1414	0.28	305.3(100), 363.2(37)	$C_{17}H_{24}O_7$	-(C5H8O)	4-acetoxy T-2 tetraol

Table S4. Metabolites of 3-AcDON generated in phase I and phase II, detected in ESI(-), as $[M+CH_3COO-H]^-$ ions, except Gluc-3AcDON which was detected as $[M-H]^-$ ion. Unless otherwise specified fragments with intensity >10% are shown in the table.

3-AcDON phase I and II metabolites								
**Name**	**RT, min**	**Measured m/z**	**Theoretical m/z**	**ppm**	**Fragments, CID**	**Formula**	**Transformation**	**Comments**
3-AcDON	5.67	397.1497	397.1505	2.01	307.2(11), 337.2(100)	$C_{17}H_{22}O_7$	parent	
DON	3.96	355.1394	355.1399	1.41	265.1(19), 295.1(100)	$C_{15}H_{20}O_6$	-(C2H2O)	Non-enzymatic
DOM-1	4.24	339.1448	339.1449	0.29	No MS2		-(C2H2O2)	Non-enzymatic
Gluc-3-AcDON	5.13	513.1613	513.1613	0	175.0(35), 191.0(26), 193.0(69), 203.1(15), 217.0(11), 229.1(10), 247.1(29), 265.2(14), 289.2(10), 307.2(100), 337.2(14), 453.1(65), 471.1(62), 495.1(66)	$C_{23}H_{30}O_{13}$	+(C6H8O6)	Gluc-3AcDON

Table S5. Metabolites of 15-AcDON generated in phase I and phase II, detected in ESI(+), as $[M+Na]^+$ ions of 15-AcDON and Gluc-15-AcDON, except DON which was detected as $[M+H]^+$ ion. Unless otherwise specified fragments with intensity >10% are shown in the table.

15-AcDON phase I and II metabolites								
**Name**	**RT, min**	**Measured m/z**	**Theoretical m/z**	**ppm**	**Fragments, CID**	**Formula**	**Transformation**	**Comments**
15-AcDON	5.57	361.1258	361.1258	0	158.1(32), 159.2(90),	$C_{17}H_{22}O_7$	parent	

					165.1(15), 167.1(14), 217.2(31), 283.2(10), 289.0(15), 301.1(100), 311.3(11), 325.4(19), 329.3(38), 343.3(82), 344.4(24)			
DON	3.96	297.1330	297.1333	1.01	NO MS2	$C_{17}H_{12}O_8$	-(C2H2O)	Non-enzymatic
Gluc-15-AcDON	5.20	537.1575	537.1579	0.74	361.47(100)	$C_{23}H_{30}O_{13}$	+(C6H8O6)	

Table S6. Metabolites of DON generated in phase I and phase II, detected in ESI(-), as [M+CH3COO-H]− ions, except Gluc-DON which was detected as [M-H]- ion. Unless otherwise specified fragments with intensity >10% are shown in the table.

DON phase I and II metabolites								
**Name**	**RT, min**	**Measured m/z**	**Theoretical m/z**	**ppm**	**Fragments, CID**	**Formula**	**Transformation**	**Comments**
DON	3.96	355.1393	355.1399	1.67	265.1(21), 295.1(100)	$C_{15}H_{20}O_6$	parent	
NIV	1.97	371.1348	371.1348	0	304.4(100)	$C_{15}H_{20}O_7$	+(O)	Non-enzymatic
peak 1-339	4.11	339.1449	339.1449	0	249.1(13), 279.1(100)	$C_{15}H_{20}O_5$	-(O)	Non-enzymatic, DOM-1
Peak 2-339	4.69	339.1449	339.1449	0	231.2(14), 249.1(100), 256.9(65), 261.2(14), 279.1(55), 321.2(24), 329.6(13)	$C_{15}H_{20}O_5$	-(O)	Non-enzymatic, DOM-1 isomer
Peak 3-339	5.08	339.1448	339.1449	0.29	No MS2	$C_{15}H_{20}O_5$	-(O)	Non-enzymatic, Low intensity

								peak, DOM-1 isomer
Peak 4-339	5.33	339.1449	339.1449	0	163.1(11), 231.1(13), 249.4(100)	$C_{15}H_{20}O_5$	-(O)	Non-enzymatic, DOM-1 isomer
Gluc-DON Peak 1	3.48	471.1508	471.1508	0	193.0(84), 265.1(50) 300.15(86), 341.11(69) 389.0(72), 410.9(81) 441.1(72), 443.9(77) 453.0(100)*	$C_{21}H_{28}O_{12}$	+(C6H8O6)	Peaks are nor resolved, 3 -Gluc-DON
Gluc-DON Peak 2	3.48	471.1508	471.1508	0	193.1(12), 265.2(15) 300.1(40), 322.8(17) 323.5(33), 341.2(25) 389.0(16), 422.7(100) 423.6(14), 441.3(16) 453.1(29), 461.8(16)	$C_{21}H_{28}O_{12}$	+(C6H8O6)	Peaks are nor resolved, 15-Gluc-DON

Table S7. Metabolites of FUS-X generated in phase I and phase II, detected in ESI(-), as [M+CH3COO-H]− ions, except Gluc-FUS-X which was detected as [M-H]- ion. Unless otherwise specified fragments with intensity >10% are shown in the table.

FUS-X phase I and II metabolites								
**Name**	**RT, min**	**Measured m/z**	**Theoretical m/z**	**ppm**	**Fragments, CID**	**Formula**	**Transformation**	**Comments**
FUS-x	4.93	413.1448	413.1454	1.33	353.3(100)	$C_{17}H_{22}O_8$	parent	
NIV	1.97	371.1347	371.1348	0.27	304.3(100.0)	$C_{15}H_{20}O_7$	-(C2H2O)	Non-enzymatic
Gluc-FUS-X	4.62	529.1561	529.1563	0.38	245.1(12), 426.1(34), 448.1(20), 469.1(100),	$C_{23}H_{30}O_{14}$	+(C6H8O6)	

					470.1(20), 487.2(34), 499.1(10), 510.8(20)			

Table S8. Metabolites of NIV generated in phase I and phase II, detected in ESI(-), as [M+CH3COO-H]− ions, except Gluc-NIV which was detected as [M-H]- ion. Unless otherwise specified fragments with intensity >10% are shown in the table.

**NIV phase I and II metabolites**								
**Name**	**RT, min**	**Measured m/z**	**Theoretical m/z**	**ppm**	**Fragments, CID**	**Formula**	**Transformation**	**Comments**
NIV	1.97	371.1343	371.1348	1.35	304.33(100.0)	$C_{15}H_{20}O_7$	-(C2H2O)	
Peak 1-355	2.5	355.1396	355.1398	0.56	217.07(44), 265.12(33), 273(11), 295.09(100)	$C_{15}H_{20}O_6$	-(O)	Non-enzymatic, DNIV
Peak 2-355	2.96	355.1396	355.1398	0.56	No MS2	$C_{15}H_{20}O_6$	-(O)	Non-enzymatic, DNIV isomer
Peak 3-355	4.28	355.1396	355.1398	0.56	No MS2	$C_{15}H_{20}O_6$	-(O)	Non-enzymatic, DNIV isomer
Gluc-NIV	1.29	487.1458	487.1457	0.18	NO MS2	$C_{21}H_{28}O_{13}$	+(C6H8O6)	
Gluc-NIV	1.56	487.1456	487.1457	0.23	352.94(100), 404.86(83), 418.91(56), 426.87(96), 468.96(60)	$C_{21}H_{28}O_{13}$	+(C6H8O6)	

Table S9. AFB1 and its metabolites of phase I reactions, detected in ESI(+), as [M+H]+ ions and * [M+Na]+ ions for AFL. Unless otherwise specified fragments with intensity >10% are shown in the table.

AFB1 phase I metabolites								
**Name**	**RT, min**	**Measured m/z**	**Theoretical m/z**	**ppm**	**Fragments, HCD**	**Formula**	**Transformation**	**Comments**
AFB1	8.10	313.0707	313.0707	0	285.0756(15), 313.0703(100)	$C_{17}H_{12}O_6$	N/A	Parent
Peak 1-329	5.03	329.0655	329.0661	1.8	206.0571(19), 283.0597(11), 301.0703(16), 311.0547(22), 329.0649(100)	$C_{17}H_{12}O_7$	+(O)	AFBO
Peak 2-329	6.32	329.0655	329.0661	1.8	259.0600(20), 273.0757(45), 301.0704(29), 311.0548(12), 329.0651(100)	$C_{17}H_{12}O_7$	+(O)	AFM1
Peak 1-347	5.63	347.0760	347.0761	0.23	259.0598(13), 273.0755(78), 283.0597(38), 287.0546(14), 289.0703(26), 301.0701(98), 311.0546(21), 329.0650(100), 347.0546(25)	$C_{17}H_{14}O_8$	+(H2 O2)	AFB1-diol/isomers
Peak 2-347	5.99	347.0761	347.0761	0	273.0757(18), 283.0599(83), 287.0548(18), 301.0705(79), 311.0549(10), 329.0653(100), 347.0759(11)	$C_{17}H_{14}O_8$	+(H2 O2)	AFB1-diol/isomers
Peak 1-299	7.05	299.0549	299.0550	0.3	271.0605(41), 299.0435(11) 299.0554(100)	$C_{16}H_{10}O_6$	-(CH2)	Low intensity

Peak 2-299	7.31	299.0549	299.0550	0.3	271.0602(11), 299.0550(100)	$C_{16}H_{10}O_6$	-(CH2)	AFP1
Peak 1-337	7.66	337.0682	337.0682*	0	No MS2	$C_{17}H_{14}O_6$	+(H2)	AFL isomer, low intensity
Peak 2-337	8.60	337.0682	337.0682*	0	No MS2	$C_{17}H_{14}O_6$	+(H2)	AFL
Peak 1-331	5.45	331.0812	331.0812	0	219.0651(10), 229.0858(10), 243.0651(42), 257.0807(33), 271.0600(16), 285.0757(71), 287.0912(12), 313.0705(100)	$C_{17}H_{14}O_7$	+(H2)+(O)	The rest peak are non-enzymatic

Table S10. AFB2 and its metabolites of phase I reactions, detected in ESI(+), as [M+H]+ ions. Unless otherwise specified fragments with intensity >10% are shown in the table.

AFB2 phase I metabolites								
**Name**	**RT, min**	**Measured m/z**	**Theoretical m/z**	**ppm**	**Fragments, CID**	**Formula**	**Transformation**	**Comments**
AFB2	7.53	315.0864	315.0863	0.32	259.1(24), 273.1(11), 285.2(10), 287.2(100), 288.2(12), 297.1(44)	$C_{17}H_{14}O_6$	parent	
Peak 1-329	4.77	331.0811	331.0813	0.60	No MS2	$C_{17}H_{14}O_7$	+(O)	Low intensity peak, non-enzymatic, AFM2
Peak 2-329	5.92	331.0812	331.0813	0.30	191.0(20), 273.1(17), 285.1(19),	$C_{17}H_{14}O_7$	+(O)	Non-enzymatic, AFQ2

					303.1(31), 313.1(100), 314.1(17)			
Peak 3-329	6.73	331.0812	331.0813	0.30	285.1(14), 303.2(27), 313.1(100), 314.1(17)	$C_{17}H_{14}O_7$	+(O)	Non-enzymatic, AB2A

Table S11. AFG1 and its metabolite, OH-AFG1, detected in ESI(+), as [M+H]+ ions. Unless otherwise specified fragments with intensity >10% are shown in the table.

**AFG1 phase I metabolites**								
**Name**	**RT, min**	**Measured m/z**	**Theoretical m/z**	**ppm**	**Fragments, CID**	**Formula**	**Transformation**	**Comments**
AFG1	7.08	329.0656	329.0656	0	243.11(11), 283.14(10), 301.15(20), 311.14(100), 312.11(10)	$C_{17}H_{12}O_7$	N/A	
Peak 1-345	5.89	345.0604	345.0605	0.3	273.16(19), 275.12(15), 289.16(36), 299.12(11), 303.15(22), 317.17(100)	$C_{17}H_{12}O_8$	+(O)	AFGM1

Table S12. AFG2 and its metabolites of phase I reactions, detected in ESI(+), as [M+H]+ ions. Unless otherwise specified fragments with intensity >10% are shown in the table.

**AFG2 phase I metabolites**								
**Name**	**RT, min**	**Measured m/z**	**Theoretical m/z**	**ppm**	**Fragments, CID**	**Formula**	**Transformation**	**Comments**
AFG2	6.73	331.0814	331.0813	0.30		$C_{17}H_{14}O_7$	parent	
Peak 1-347	5.47	347.0760	347.0761	0.29	No MS2	$C_{17}H_{14}O_8$	+(O)	Low intensity peak, non-enzymatic
Peak 2-347	5.55	347.0760	347.0761	0.29	No MS2	$C_{17}H_{14}O_8$	+(O)	Low intensity peak, non-enzymatic
Peak 3-347	5.67	347.0760	347.0761	0.29	No MS2	$C_{17}H_{14}O_8$	+(O)	Low intensity

								peak, non-enzymatic, AFGM2
Peak 4-347	5.87	347.0760	347.0761	0.29	No MS2	$C_{17}H_{14}O_8$	+(O)	Low intensity peak, non-enzymatic, AFG2A

Table S13. Metabolites of ZEN generated in phase I and phase II, detected in ESI(-), as [M-H]− ions. Unless otherwise specified fragments with intensity >10% are shown in the table.

ZEN phase I metabolites								
**Name**	**RT, min**	**M-H measured**	**M-H theoretical**	**ppm**	**Fragments, CID**	**Formula**	**Transformation**	**Comment**
ZEN-main	13.52	317.1394	317.1394	0	149.1(10), 175.1(10), 273.5(100), 299.4(80)	$C_{18}H_{22}O_5$	parent	
ZEN-MINOR	13.15	317.1394	317.1394	0	149.1(22), 161.1(10), 175.1(15), 203.1(30), 273.2(90), 299.2(100)	$C_{18}H_{22}O_5$	N/A	isomer
β-ZOL	12.30	319.1547	319.1545	0.6	275.2(100), 287.8(10), 299.1(25), 301.1(70)	$C_{18}H_{24}O_5$	+(H2)	
ZAN	13.27	319.1544	319.1545	0.3	205.1(10), 275.4(100), 301.1(20)	$C_{18}H_{24}O_5$	+(H2)	
α-ZOL	13.48	319.1550	319.1545	1.6	No ms2	$C_{18}H_{24}O_5$	+(H2)	
Peak 1-331	8.69	331.1186	331.1182	1.2	202.1(40), 287.2(80), 303.1(100), 312.2(60)	$C_{18}H_{20}O_6$	-(H2)+(O)	13-OH-ZEN-quinone
Peak 1-335	7.53	335.1500	335.1495	1.5	211.0(30), 253.0(50), 291.2(100), 315.0(70), 317.1(100)	$C_{18}H_{24}O_6$	+(H2+(O)	8-OH-α or β-ZOL
Peak 2-335	12.23	335.1496	335.1495	0.3	No ms2	$C_{18}H_{24}O_6$	+(H2+(O)	13-OH-α-ZOL
Peak 1-333	8.11	333.1342	333.1338	1.2	289.1(80), 305.2(100), 315.1(40)	$C_{18}H_{22}O_6$	+(O)	α or β-OH-ZOL-quinone

Peak 2-333	8.71	333.1340	333.1338	0.6	288.2(70), 289.2(100), 304.2(90), 315.1(30)	$C_{18}H_{22}O_6$	+(O)	α or β-OH-ZOL-quinone
Peak 3-333	8.87	333.1341	333.1338	0.9	No ms2	$C_{18}H_{22}O_6$	+(O)	2 or 3-OH-ZEN
Peak 4-333	9.38	333.1342	333.1338	1.2	250.1(20), 289.1(100), 315.1(80)	$C_{18}H_{22}O_6$	+(O)	6 or 8-OH-ZEN
Peak 5-333	10.96	333.1341	333.1338	0.9	216.1(15), 289.2(100), 314.3(60), 315.2(60)	$C_{18}H_{22}O_6$	+(O)	6 or 8-OH-ZEN
Peak 6-333	11.52	333.1341	333.1338	0.9	191.0(60), 289.2(90), 314.3(100), 315.2(90)	$C_{18}H_{22}O_6$	+(O)	4-OH-ZEN or 5-OH-ZEN or 9-OH-ZEN
Peak 7-333	12.22	333.1342	333.1338	1.2	289.3(20), 315.5(100)	$C_{18}H_{22}O_6$	+(O)	10-OH-ZEN
Peak 8-333	12.51	333.134	333.1338	0.6	175.1(15), 203.1(30), 289.2(50), 315.4(100)	$C_{18}H_{22}O_6$	+(O)	15-OHZEN
Peak 9-333	12.62	333.1342	333.1338	1.2	No ms2	$C_{18}H_{22}O_6$	+(O)	13-OH-ZEN
**ZEN phase II metabolites**								
**Name**	**RT, min**	**M-H measured**	**M-H theoretical**	**ppm**	**Fragments, CID**	**Formula**	**Transformation**	**Comment**
Peak 1-493	5.82	493.1714	493.1710	0.8	175.0(15), 317.2(100), 410.9(10), 449.2(31), 475.0(11)	$C_{24}H_{30}O_{11}$	+(C6H8O6)	16-Gluc-ZEN
Peak 2-493	7.20	493.1714	493.1710	0.8	175.0(20), 317.2(100)	$C_{24}H_{30}O_{11}$	+(C6H8O6)	14-Gluc-ZEN
Peak 3-493	12.22	493.1714	493.1710	0.8	174.9(20), 316.3(15), 317.2(100), 411.0(25), 433.0(14), 473.0(15), 474.3(17), 475.1(17)	$C_{24}H_{30}O_{11}$	+(C6H8O6)	Shallow peak
Peak 1-495	5.57	495.1869	495.1866	0.6	175.1(20), 319.2(100), 397.0(10), 413.11(90), 413.9(10), 433.2(10), 434.9(46), 440.1(12), 463.1(18), 473.6(16), 477.1(58), 478.0(26), 479.0(10,) 485.8(38), 486.5(10)	$C_{24}H_{32}O_{11}$	+(C6H10O6)	16-Gluc-β-ZOL

Peak 2-495	5.89	495.18697	495.1866	0.7	175.0(18), 317.2(10), 319.2(100), 331.1(10), 397.1(32), 413.0(78), 433.1(10), 433.9(30), 451.1(24), 454.7(14), 464.2(10), 465.2(20), 475.0(10), 476.9(65), 477.9(10) 486.1(25)	$C_{24}H_{32}O_{11}$	+(C6H10O6)	14-Gluc-β-ZOL or 16-Gluc-ZAN,
Peak 3-495	7.31	495.1858	495.1866	1.6	175.0(42), 176.0(10), 317.2(40), 318.2(82), 319.2(100)	$C_{24}H_{32}O_{11}$	+(C6H10O6)	14-Gluc-α-ZOL
Peak 4-495	12.22	495.1866	495.1866	0.0	319.2(25), 413.0(22), 435.0(10), 451.2(100), 475.0(16), 477.2(17)	$C_{24}H_{32}O_{11}$	+(C6H10O6)	7-Gluc-α-ZOL
Peak 1-509	6.43	509.1662	509.1659	0.6	175.0(10), 333.2(100), 427.0(10), 490.9(10)	$C_{24}H_{30}O_{12}$	+(C6H8O7)	Gluc-15-OH-ZEN
Peak 2-509	7.54	509.1660	509.1659	0.2	332.4(10), 333.2(100), 491.1(12)	$C_{24}H_{30}O_{12}$	+(C6H8O7)	Gluc-13-OH-ZEN
Peak 1-669	5.28	669.2035	669.2031	0.6	493.1(100)	$C_{24}H_{30}O_{12}$	+(C12H16O12)	2xGluc-ZEN

Table S14. Metabolites of *α*-ZOL generated in phase I and phase II, detected in ESI(-), as [M-H]− ions. Unless otherwise specified fragments with intensity >10% are shown in the table.

**α-ZOL phase I metabolite**								
**Name**	**RT, min**	**M-H measured**	**M-H theoretical**	**ppm**	**Fragments, CID**	**Formula**	**Transformation**	**Comment**
α-ZOL	13.63	319.1549	319.1545	1.3	257.2(4), 275.6(100), 301.5(80)	$C_{18}H_{24}O_5$	parent	
β-ZOL	12.41	319.1549	319.1545	1.3	275.5(100), 301.2(35)	$C_{18}H_{24}O_5$	isomer	Non enzymatic
One more isomer	13.01	319.1549	319.1545	1.3	275.5(100), 301.2(10)	$C_{18}H_{24}O_5$	isomer	Non enzymatic
ZEN	13.67	317.1394	317.1394	0	149.1(11), 175.1(10), 261.2(5), 273.5(100), 299.4(80)	$C_{18}H_{22}O_5$	-(H2)	
Peak 1-331	9.05	331.1186	331.1182	1.2	287.2 (70), 303.2(100), 312.3(10), 313.2(15)	$C_{18}H_{20}O_6$	-(H4)+(O)	

Peak 1-333	8.36 main	333.1343	333.1338	1.5	261.3(12), 289.3(68), 305.2(100), 315.2(10)	$C_{18}H_{22}O_6$	-(H2)+(O0	
Peak 2-333	9.69	333.1342	333.1338	1.2	No ms2	$C_{18}H_{22}O_6$	-(H2)+(O)	
Peak 3-333	11.94	333.1343	333.1338	1.5	190.0 (10), 191.0(82), 201.0(10), 219.1(10), 261.1(26), 289.2(100), 305.2(13), 314.2(20), 315.2(45)	$C_{18}H_{22}O_6$	-(H2)+(O)	
Peak 4-333	12.33	333.1342	333.1338	1.2	191.0(40), 197.1(10), 271.3(10), 289.2(35), 313.1(12), 314.3(11), 315.2(100)	$C_{18}H_{22}O_6$	-(H2)+(O)	
Peak 5-333	12.63	333.1343	333.1338	1.5	175.1(18), 191.1(24), 203.0 (28), 216.1(13), 271.24(20), 289.2(100), 313.1(21), 314.2(24), 315.2(95)	$C_{18}H_{22}O_6$	-(H2)+(O)	
Peak 6-333	12.72	333.1341	333.1338	0.9	No ms2	$C_{18}H_{22}O_6$	-(H2)+(O)	
Peak 1-335	7.70	335.1499	335.1495	1.2	No ms2	$C_{18}H_{24}O_6$	+(O)	
Peak 2-335	9.33	335.1499	335.1495	1.2	No ms2	$C_{18}H_{24}O_6$	+(O)	
Peak 3-335	11.31	335.1498	335.1495	0.9	161.0(14), 163.0(18), 190.0(18), 235.2(10), 273.2(22), 291.2(100), 317.2(52)	$C_{18}H_{24}O_6$	+(O)	
Peak 4-335	11.86	335.1499	335.1495	1.2	190.0(30), 203.1(10), 219.1(10), 291.2(100), 292.2(15), 307.2(12), 317.2(20)	$C_{18}H_{24}O_6$	+(O)	
Peak 5-335	12.34	335.1498	335.1495	0.9	163.1(10), 175.1(11), 189.1(34), 273.2(19), 291.2(100), 299.2(19), 315.0(14), 317.2(82)	$C_{18}H_{24}O_6$	+(O)	
Peak 6-335	12.44	335.1499	335.1495	1.2	175.0(84), 179.1(24), 190.0(10), 247.2(10), 273.23(24), 291.2(80), 317.2(100)	$C_{18}H_{24}O_6$	+(O)	
Peak 7-335	12.71	335.1498	335.1495	0.9	No ms2	$C_{18}H_{24}O_6$	+(O)	
Peak 8-335	13.18	335.1500	335.1495	1.5	175.0(40), 207.1(22), 247.2(22), 299.2(16), 317.3(100)	$C_{18}H_{24}O_6$	+(O)	

Peak 3-511	7.63	511.1814	511.1816	0.4	335.2(100), 493.2(11)	$C_{24}H_{32}O_{12}$	+(C6H8O7)	Gluc-(+(O))
Peak 4-511	8.94	511.1820	511.1816	0.8	175.1(16), 192.2(28), 317.3(18), 335.3(100), 347.1(57), 393.3(11), 397.0(10), 429.1(49), 451.0(14), 467.1(39), 493.1(34)	$C_{24}H_{32}O_{12}$	+(C6H8O7)	Gluc-(+(O))
Peak 1-671	5.31	671.2191	671.2187	0.6	495.2(100)	$C_{30}H_{40}O_{17}$	+C12H16O12	di-Gluc-α-ZAL
**α-ZOL phase II metabolite**								
**Name**	**RT, min**	**M-H measured**	**M-H theoretical**	**ppm**	**Fragments, CID**	**Formula**	**Transformation**	**Comment**
Peak 1-493	5.95	493.1714	493.1710	0.8	No MS2	$C_{24}H_{30}O_{11}$	+(C6H6O6)	Gluc-ZEN
Peak 2-493	7.44	493.1721	493.1710	2.2	175.0(22), 317.2(100), 411.1(10)	$C_{24}H_{30}O_{11}$	+(C6H6O6)	Gluc-ZEN
Peak 1-495	5.66	495.1870	495.1866	0.8	175.1(20), 319.2(100), 451.2(36), 477.2(12)	$C_{24}H_{32}O_{11}$	+(C6H8O6)	16-Gluc-α-ZOL
Peak 2-495	7.53	495.1871	495.1866	1.0	175.0(32), 319.2(100)	$C_{24}H_{32}O_{11}$	+(C6H8O6)	14-Gluc-α-ZOL
Peak 3-495	12.37	495.1869	495.1866	0.6	451.5(100)	$C_{24}H_{32}O_{11}$	+(C6H8O6)	7-Gluc-α-ZOL
Peak 1-511	6.01	511.1819	511.1816	0.6	No ms2	$C_{24}H_{32}O_{12}$	+(C6H8O7)	Gluc-(+(O))
Peak 2-511	6.51	511.1819	511.1816	0.6	No ms2	$C_{24}H_{32}O_{12}$	+(C6H8O7)	Gluc-(+(O))
Peak 3-511	7.63	511.1814	511.1816	0.4	335.2(100), 493.2(11)	$C_{24}H_{32}O_{12}$	+(C6H8O7)	Gluc-(+(O))
Peak 4-511	8.94	511.1820	511.1816	0.8	175.1(16), 192.2(28), 317.3(18), 335.3(100), 347.1(57), 393.3(11), 397.0(10), 429.1(49), 451.0(14), 467.09(39), 493.1(34)	$C_{24}H_{32}O_{12}$	+(C6H8O7)	Gluc-(+(O))
Peak 1-671	5.31	671.2191	671.2187	0.6	495.2(100)	$C_{30}H_{40}O_{17}$	+C12H16O12	di-Gluc-α-ZAL

Table S15. Metabolites of β-ZOL generated in phase I and phase II, detected in ESI(-), as [M-H]− ions. Unless otherwise specified fragments with intensity >10% are shown in the table.

β-ZOL phase II metabolite								
**Name**	**RT, min**	**M-H measured**	**M-H theoretical**	**ppm**	**Fragments, CID**	**Formula**	**Transformation**	**Comment**
β-ZOL	12.42	319.1547	319.1545	0.6	275.2(100), 301.5(80)	$C_{18}H_{24}O_5$	parent	
α-ZOL	13.62	319.1552	319.1545	2.2	274.3(24), 275.5(100), 300.2(12), 301.2(26)	$C_{18}H_{24}O_5$	isomer	Non-enzymatic
ZEN	13.67	317.1396	317.1394	0.6	149.1(11), 175.1(10), 273.46(100), 299.38(85)	$C_{18}H_{22}O_5$	-(H2)	
Peak 1-335	7.55	335.1499	335.1495	1.2	190.0(28), 291.2(100), 292.2(11), 307.2(10), 317.2(15)	$C_{18}H_{24}O_6$	+(O)	
Peak 2-335	8.68	335.1499	335.1495	1.2	175.0(100), 273.2(10), 291.2(15), 317.2(15)	$C_{18}H_{24}O_6$	+(O)	Only in heated
Peak 3-335	8.90	335.1499	335.1495	1.2	No ms2	$C_{18}H_{24}O_6$	+(O)	
Peak 4-335	11.64	335.1499	335.1495	1.2	175.0(100), 273.2(10), 291.2(30), 315.1(10), 317.2(24)	$C_{18}H_{24}O_6$	+(O)	
Peak 5-335	12.03	335.1499	335.1495	1.2	No ms2	$C_{18}H_{24}O_6$	+(O)	
Peak 6-335	12.17	335.1499	335.1495	1.2	193.1 (40), 273.3(15), 291.2(35), 315.07(10), 317.3(100)	$C_{18}H_{24}O_6$	+(O)	
Peak 7-335	12.35	335.1499	335.1495	1.2	No ms2	$C_{18}H_{24}O_6$	+(O)	
Peak 1-333	8.43	333.1343	333.1338	1.5	261.2(15), 289.2(73), 305.2(100), 315.2(12)	$C_{18}H_{22}O_6$	-(H2)+(O)	
Peak 1-331	9.16	331.1186	331.1182	1.2	No ms2	$C_{18}H_{20}O_6$	-(H4)+(O)	
**β-ZOL phase II metabolite**								
**Name**	**RT**	**M-H measured**	**M-H theoretical**	**ppm**	**Fragments, CID**	**Formula**	**Transformation**	**Comment**
Peak 1-495	5.57	495.1871	495.1866	1.0	175.0(20), 319.2(100), 451.3(27), 477.2(10)	$C_{24}H_{32}O_{11}$	+(C6 H8 O6)	16-Gluc-β-ZOL

Peak 2-495	6.00	495.1871	495.1866	1.0	175.0(28), 319.2(100), 451.3(14)	$C_{24}H_{32}O_{11}$	+(C6 H8 O6)	14-Gluc-β-ZOL
Peak 3-495	8.96	495.1871	495.1866	1.0	407.3(11), 451.3(100), 477.2(15)	$C_{24}H_{32}O_{11}$	+(C6 H8 O6)	7-Gluc-β-ZOL
Peak 1-493	5.96	493.1718	493.1710	1.6	No ms2	$C_{24}H_{30}O_{11}$	+(C6H6O6)	
Peak 2-493	7.59	493.1714	493.1710	0.8	175.0(23), 317.2(100), 411.0(22), 432.92(10)	$C_{24}H_{30}O_{11}$	+(C6H6O6)	
Peak 1-511	5.86	511.1819	511.1816	0.6	335.4(100)	$C_{24}H_{32}O_{12}$	+(C6H8O7)	
Peak 1-671	5.15	671.2191	671.2187	0.6	495.4(100)	$C_{30}H_{40}O_{17}$	+(C12H16O12)	di-Gluc-β-ZOL

Table S16. Metabolites of ZAN generated in phase I and phase II, detected in ESI(-), as [M-H]− ions. Unless otherwise specified fragments with intensity >10% are shown in the table.

ZAN phase I metabolites								
**Name**	**RT, min**	**M-H measured**	**M-H theoretical**	**ppm**	**Fragments, CID**	**Formula**	**Transformation**	**Comment**
ZAN	13.35	319.1545	319.1545	0.0	205.2(26), 275.5(100), 301.5(70)	$C_{18}H_{24}O_5$	parent	
β-ZAL	11.33	321.1707	321.1702	1.6	277.5(100), 303.2(20)	$C_{18}H_{26}O_5$	+(H2)	
α-ZAL	12.92	321.1707	321.1702	1.6	277.6(100), 303.5(86)	$C_{18}H_{26}O_5$	+(H2)	
Peak 1-333	9.14	333.1341	333.1338	0.9	289.5(92), 305.5(100), 315.2(12)	$C_{18}H_{22}O_6$	-(H2)+(O)	
Peak 1-335	8.69	335.1497	335.1495	0.6	291.2(30), 307.5(100)	$C_{18}H_{24}O_6$	+(O)	
Peak 2-335	9.06	335.1497	335.1495	0.6	290.3(38), 291.2(34), 306.4(100), 307.2(18), 317.2(16)	$C_{18}H_{24}O_6$	+(O)	
Peak 3-335	9.41	335.1497	335.1495	0.6	290.4(10), 291.5(100), 306.2(12), 317.2(12)	$C_{18}H_{24}O_6$	+(O)	
Peak 4-335	11.05 no peak	335.1498	335.1495	0.9	291.2(45), 317.2(100)	$C_{18}H_{24}O_6$	+(O)	

Peak 5-335	12.11, main	335.1498	335.1495	0.9	193.1(12), 221.1(10), 273.2(14), 291.2(100), 307.2(14), 317.18(85)	$C_{18}H_{24}O_6$	+(O)	
Peak 1-337	6.47	337.1656	337.1651	1.5	No MS2	$C_{18}H_{26}O_6$	+(H2)+(O)	
Peak 2-337	6.83	337.1656	337.1651	1.5	177.1(14), 231.2(12), 275.2(24), 293.2(100) 319.2(16)	$C_{18}H_{26}O_6$	+(H2)+(O)	
Peak 3-337	7.45	337.1655	337.1651	1.2	No MS2	$C_{18}H_{26}O_6$	+(H2)+(O)	
Peak 4-337	7.65	337.1656	337.1651	1.5	No MS2	$C_{18}H_{26}O_6$	+(H2)+(O)	
Peak 5-337	9.78	337.1656	337.1651	1.5	177.1(10), 231.1(8), 275.2(14), 293.4(100), 319.2(10)	$C_{18}H_{26}O_6$	+(H2)+(O)	
Peak 6-337	10.31	337.1656	337.1651	1.5	No MS2	$C_{18}H_{26}O_6$	+(H2)+(O)	
Peak 7-337	11.74 main	337.1656	337.1651	1.5	177.0(16), 275.2(22), 293.3(100) 319.22(20)	$C_{18}H_{26}O_6$	+(H2)+(O)	
Peak 8-337	12.20	337.1653	337.1651	0.6	No MS2	$C_{18}H_{26}O_6$	+(H2)+(O)	
Peak 1-349	6.44	349.1291	349.1287	1.1	No MS2	$C_{18}H_{22}O_7$	-(H2)+(O2)	
Peak 2-349	6.86	349.1291	349.1287	1.1	163.0(20), 177.0(45), 179.1(15), 191.1(48), 217.1(44), 235.1(70), 261.2(11), 267.1(10), 277.2(22), 287.2(41), 303.2(11), 305.2(100), 321.2(98), 330.4(16), 331.2(68), 339.7(10)	$C_{18}H_{22}O_7$	-(H2)+(O2)	
Peak 1-351	6.34	351.1447	351.1444	0.9	No MS2	$C_{18}H_{24}O_7$	+(O2)	
Peak 2-351	6.79	351.1447	351.1444	0.9	191.0(10), 205.0(14), 269.0(15), 279.2(20), 289.2(11), 307.2(100), 323.2(70), 333.1(32)	$C_{18}H_{24}O_7$	+(O2)	
Peak 3-351	7.15	351.1447	351.1444	0.9	No MS2	$C_{18}H_{24}O_7$	+(O2)	
Peak 4-351	7.62	351.1448	351.1444	1.1	219.1(18), 307.3(28), 333.2(100)	$C_{18}H_{24}O_7$	+(O2)	
Peak 5-351	8.28	351.1448	351.1444	1.1	289.2(24), 307.3(100), 333.2(68)	$C_{18}H_{24}O_7$	+(O2)	

Peak 6-351	9.06	351.1447	351.1444	0.9	269.0(10), 289.2(15), 307.3(40), 315.3(16), 333.3(100)	$C_{18}H_{24}O_7$	+(O2)	Low intensity peak
**ZAN phase II metabolites**								
**Fragments**	**Formula**	**Transformation**	**Comment**	**ppm**	**Fragments, CID**	**Formula**	**Transformation**	**Comment**
Peak 1-497	5.70	497.2026	497.2023	0.6	175.0(25), 321.2(100), 453.2(30), 479.1(15)	$C_{24}H_{34}O_{11}$	+(C6H10O6)	
Peak 2-497	5.90	497.2025	497.2023	0.4	175.0(50), 321.2(100), 452.2(10)	$C_{24}H_{34}O_{11}$	+(C6H10O6)	
Peak 3-497	6.53	497.2026	497.2023	0.6	175.0(40), 321.2(100)	$C_{24}H_{34}O_{11}$	+(C6H10O6)	
Peak 4-497	10.25	497.2025	497.2023	0.4	321.2(10), 415.2(10), 453.2(100), 479.2(24)	$C_{24}H_{34}O_{11}$	+(C6H10O6)	
Peak 1-495	5.96	495.1872	495.1866	1.2	175.0(20), 319.2(100), 451.2(28), 477.2(10)	$C_{24}H_{32}O_{11}$	+(C6H8O6)	
Peak 2-495	7.18	495.1869	495.1866	0.6	175.0(30), 319.2(100), 477.2(5)	$C_{24}H_{32}O_{11}$	+(C6H8O6)	
Peak 1-513	5.07	513.1975	513.1972	0.6	No MS2	$C_{24}H_{34}O_{12}$	+(C6H10O7)	
Peak 2-513	5.55	513.1974	513.1972	0.4	No MS2	$C_{24}H_{34}O_{12}$	+(C6H10O7)	
Peak 3-513	5.90	513.1973	513.1972	0.2	175.0(20), 337.2(100)	$C_{24}H_{34}O_{12}$	+(C6H10O7)	
Peak 4-513	8.12	513.1974	513.1972	0.4	No MS2	$C_{24}H_{34}O_{12}$	+(C6H10O7)	
Peak 5-513	8.45	513.1974	513.1972	0.4	175.0(2), 337.2(100), 469.2(40), 495.2(15),	$C_{24}H_{34}O_{12}$	+(C6H10O7)	
Peak 1-511	5.31-5.41	511.1818	511.1816	0.4	No MS2	$C_{24}H_{32}O_{12}$	+(C6H8O7)	
Peak 2-511	5.58	511.1818	511.1816	0.4	No MS2	$C_{24}H_{32}O_{12}$	+(C6H8O7)	
Peak 3-511	5.98	511.1818	511.1816	0.4	175.0(20), 335.2(100), 493.2(10)	$C_{24}H_{32}O_{12}$	+(C6H8O7)	

Peak 4-511	6.50	511.1818	511.1816	0.4	335.2(100)	$C_{24}H_{32}O_{12}$	+(C6H8O7)	
Peak 5-511	6.55	511.1814	511.1816	0.4	335.2(100)	$C_{24}H_{32}O_{12}$	+(C6H8O7)	
Peak 6-511	9.41	511.1819	511.1816	0.6	335.2(100), 493.2(10)	$C_{24}H_{32}O_{12}$	+(C6H8O7)	
Peak 1-671	5.80	671.2189	671.2187	0.3	495.4(100)	C30H40O 17	+(C12H16O12)	di-Gluc-ZAN
Peak 1-673	5.39	673.2344	673.2344	0	497.4(100)	C30H42O 17	+(C12H18O12)	di-Gluc-α-ZAL
Peak 1-687	5.16	687.2139	687.2136	0.44	No MS2	C30H40O 18	+(C12H16O13)	di-Gluc-(+O))
Peak 2-687	6.17	687.2139	687.2136	0.44	No MS2	C30H40O 18	+(C12H16O13)	di-Gluc-(+O))
Peak 1-689	5.07	689.2296	689.2293	0.4	No MS2	C30H42O 18	+(C12H18 O13)	di-Gluc-(+(H2)+(O))
Peak 2-689	5.69	689.2296	689.2293	0.4	No MS2	C30H42O 18	+(C12H18 O13)	di-Gluc-(+(H2)+(O))

Table S17. Metabolites of *α*-ZAL generated in phase I and phase II, detected in ESI(-), as [M-H]− ions. Unless otherwise specified fragments with intensity >10% are shown in the table.

**α-ZAL phase I metabolite**								
**Name**	**RT, min**	**M-H measured**	**M-H theoretical**	**ppm**	**Fragments, CID**	**Formula**	**Transformation**	**Comment**
α-ZAL	12.91	321.1704	321.1702	0.6	277.5(100), 303.5(85)	$C_{18}H_{26}O_5$	parent	
β-ZAL	11.33	321.1707	321.1702	1.6	277.5(100), 303.2(20)	$C_{18}H_{26}O_5$	isomer	Non enzymatic
ZAN	13.34	319.15460	319.1545	0.3	205.2(19), 275.5(100), 301.2(25)	$C_{18}H_{24}O_5$	-(H2)	
Peak 1-333	9.18	333.1344	333.1338	1.8	289.2(80), 305.2(100), 315.1(20)	$C_{18}H_{22}O_5$	-(H4)+(O)	

Peak 1-335	8.74	335.1499	335.1495	1.2	263.3(10), 291.3(75), 307.5(100), 317.8(15)	$C_{18}H_{24}O_6$	-(H2)+(O)	
Peak 2-335	11.11	335.1499	335.1495	1.2	291.2(50), 307.2(15), 315.1(12), 317.2(100)	$C_{18}H_{24}O_6$	-(H2)+(O)	
Peak 3-335	12.11	335.1499	335.1495	1.2	291.5(100), 317.5(75)	$C_{18}H_{24}O_6$	-(H2)+(O)	
Peak 1-337	6.83	337.1657	337.1651	1.8	177.1(15), 231.1(10), 275.2(20), 293.2(100), 319.2(20)	$C_{18}H_{26}O_6$	+O	found only in heated
Peak 2-337	10.34	337.1657	337.1651	1.8	293.4(100), 319.2(27)	$C_{18}H_{26}O_6$	+O	found only in heated
Peak 3-337	11.75	337.1657	337.1651	1.8	177.1(15), 255.2(10) 275.2(20), 293.2(100), 319.2(24)	$C_{18}H_{26}O_6$	+O	
Peak 4-337	12.21	337.1657	337.1651	1.8	177.1(6), 221.1(14), 293.5(100), 318.3(10), 319.2(20)	$C_{18}H_{26}O_6$	+O	
Peak 1-349	6.85	349.1293	349.1287	1.7	No MS2	$C_{18}H_{22}O_7$	-(H4) +(O2)	
Peak 1-351	6.78	351.1449	351.1444	1.4	No MS2	$C_{18}H_{24}O_7$	-(H2) +(O2)	
**α-ZAL phase II metabolite**								
**Name**	**RT, min**	**M-H measured**	**M-H theoretical**	**ppm**	**Fragments, CID**	**Formula**	**Transformation**	**Comment**
Peak 1-497	5.70	497.2027	497.2023	0.8	175.0(25), 321.2(100), 453.2(25), 479.2(10)	$C_{24}H_{34}O_{11}$	+(C6H8O6)	May be 16-Gluc-β-ZAL
Peak 2-497	5.89	497.2026	497.2023	0.6	175.0(40), 321.2(100), 441.2(14), 453.20(15), 479.1(20)	$C_{24}H_{34}O_{11}$	+(C6H8O6)	Then it is 16-Gluc-α-ZAL
Peak 3-497	6.56	497.2026	497.2023	0.6	175.0(35), 321.2(100)	$C_{24}H_{34}O_{11}$	+(C6H8O6)	14-Gluc-α-ZAL
Peak 4-497	10.19	497.2026	497.2023	0.6	453.3(100), 479.2(15)	$C_{24}H_{34}O_{11}$	+(C6H8O6)	7-Gluc-α-ZAL
Peak 5-497	12.10	497.2026	497.2023	0.6	321.2(10), 453.5(100), 479.2(30), 498.3(10)	$C_{24}H_{34}O_{11}$	+(C6H8O6)	Shallow peak
Peak 1-495	5.94	495.1871	495.1866	1.0	No MS2	$C_{24}H_{32}O_{11}$	+(C6H6O6)	16-Gluc-ZAN

Peak 2-495	7.34*	495.1871	495.1866	1.0	175.0(30), 319.2(100), 413.1(15), 477.1(14)	$C_{24}H_{32}O_{11}$	+(C6H6O6)	14-Gluc-ZAN
Peak 1 to 3 - 511	5.99, 7.39, 9.36	511.1819	511.1816	0.6	No MS2, low intensity	$C_{24}H_{32}O_{12}$	+(C6H6O7)	Gluc+(O)-(H2)
Peak 1-513	5.08	513.1975	513.1972	0.6	No MS2	$C_{24}H_{34}O_{12}$	+(C6H8O7)	Gluc+(O)
Peak 2-513	5.89 - 6.1	513.1975	513.1972	0.6	No MS2	$C_{24}H_{34}O_{12}$	+(C6H8O7)	Gluc+(O)
Peak 3-513	8.45	513.1975	513.1972	0.6	337.2(100), 469.2(28), 495.1(15)	$C_{24}H_{34}O_{12}$	+(C6H8O7)	Gluc+(O)
Peak 1- 671	5.72	671.2192	671.2187	0.7	No ms2, ow intensity peak	$C_{30}H_{40}O_{17}$	+(C12H14O12)	di-Gluc-ZAN
Peak 1- 673	5.38	673.2345	673.2344	0.1	497.4(100)	$C_{30}H_{42}O_{17}$	+(C12H16O12)	di-Gluc-α-ZAL

Table S18. Metabolites of β-ZAL generated in phase I and phase II, detected in ESI(-), as [M-H]− ions. Unless otherwise specified fragments with intensity >10% are shown in the table.

**β-ZAL phase I metabolites**								
**Name**	**RT, min**	**M-H measured**	**M-H theoretical**	**ppm**	**Fragments, CID**	**Formula**	**Transformation**	**Comment**
β-ZAL	11.48	321.1748	321.1702		277.5(100), 293.3(4), 303.4(60)	$C_{18}H_{26}O_5$	parent	
α-ZAL	12.94	321.1707	321.1702	1.6	277.6(100), 303.2(35)	$C_{18}H_{26}O_5$	isomer	
ZAN	13.39	319.1549	319.1545	1.3	205.2(25), 275.5(100), 301.4(75)	$C_{18}H_{24}O_5$	-(H2)	
Peak1-333	9.17	333.1343	333.1338	1.5	289.2(85), 305.2(100), 315.1(28)	$C_{18}H_{22}O_6$	-(H4)+(O)	13-OH_ZAN quinone
Peak 1-335	7.89	335.1499	335.1495	1.2	123.1(12), 175.1(22), 219.1(20), 253.0(30), 273.3(15), 291.2(100), 303.1(18), 307.2(20), 315.2(35), 317.1(40)	$C_{18}H_{24}O_6$	-(H2)+(O)	
Peak 2-335	8.78	335.1499	335.1495	1.2	263.2(12), 291.2(85), 307.2(100), 317.2(20)	$C_{18}H_{24}O_6$	-(H2)+(O)	13-OH-α-ZAL quinone

Peak 3-335	11.16	335.1498	335.1495	0.9	211.0(10), 253.0(22), 291.2(65), 292.2(10), 307.29(24), 315.1(24), 317.2(100), 318.2(10)	$C_{18}H_{24}O_6$	-(H2)+(O)	15-OH-ZAN
Peak 4-335	12.13	335.1499	335.1495	1.2	291.5(100), 317.5(98)	$C_{18}H_{24}O_6$	-(H2)+(O)	13-OH-ZAN
Peak 1-337	5.78	337.1656	337.1651	1.5	No MS2	$C_{18}H_{26}O_6$	+(O)	
Peak 2-337	6.49	337.1656	337.1651	1.5	177.1(12), 221.1(30), 275.2(15), 293.2(100), 319.2(20)	$C_{18}H_{26}O_6$	+(O)	
Peak 3-337	7.50	337.1656	337.1651	1.5	177.1(19), 255.2(15), 275.2(23), 293.2(100), 319.19(15)	$C_{18}H_{26}O_6$	+(O)	
Peak 4-337	7.67	337.1656	337.1651	1.5	293.5(100), 319.3(30)	$C_{18}H_{26}O_6$	+(O)	
Peak 5-337	8.13	337.1656	337.1651	1.5	No MS2	$C_{18}H_{26}O_6$	+(O)	
Peak 6-337	9.25	337.1655	337.1651	1.2	221.1(15), 293.5(100), 319.2(22)	$C_{18}H_{26}O_6$	+(O)	
Peak 7-337	9.88	337.1655	337.1651	1.2	No MS2	$C_{18}H_{26}O_6$	+(O)	
Peak 8-337	11.88	337.1655	337.1651	1.2	No MS2	$C_{18}H_{26}O_6$	+(O)	
Peak 1-349	6.83	349.1292	349.1287	1.4	No MS2	$C_{18}H_{26}O_6$	-(H4) +(O2)	
Peak 1-351	6.74	351.1449	351.1444	1.4	No MS2	$C_{18}H_{26}O_6$	-(H2) +(O2)	
**β-ZAL phase II metabolites**								
**Name**	**RT, min**	**M-H measured**	**M-H theoretical**	**ppm**	**Fragments, CID**	**Formula**	**Transformation**	**Comment**
Peak 1-497	5.67	497.2027	497.2023	0.8	175.0(30), 321.3(100), 453.3(30),479.2(10)	$C_{24}H_{34}O_{11}$	+(C6H8O6)	16-Gluc-β-ZAL
Peak 2-497	5.86	497.2027	497.2023	0.8	175.0(42), 321.3(100)	$C_{24}H_{34}O_{11}$	+(C6H8O6)	14-Gluc-β-ZAL
Peak 3-497	7.67	497.2028	497.2023	1.0	321.2(18), 403.2(14), 409.3(22), 453.2(100), 479.2(42)	$C_{24}H_{34}O_{11}$	+(C6H8O6)	7-Gluc-β-ZAL
Peak 1-495	7.31*	495.1871	495.1866	1.0	No MS2	$C_{24}H_{32}O_{11}$	+(C6H6O6)	14-Gluc-ZAN

Peak 1-513	5.71	513.1975	513.1972	0.6	No MS2	$C_{24}H_{34}O_{12}$	+(C6H8O7)	Gluc+(O)
Peak 2-513	6.29	513.1976	513.1972	0.8	337.2(100), 495.2(12)	$C_{24}H_{34}O_{12}$	+(C6H8O7)	Gluc+(O)
Peak 1-673	5.42	673.2342	673.2344	0.3	497.2(100)	$C_{30}H_{42}O_{17}$	+(C12H16O12)	di-Gluc-β-ZAL

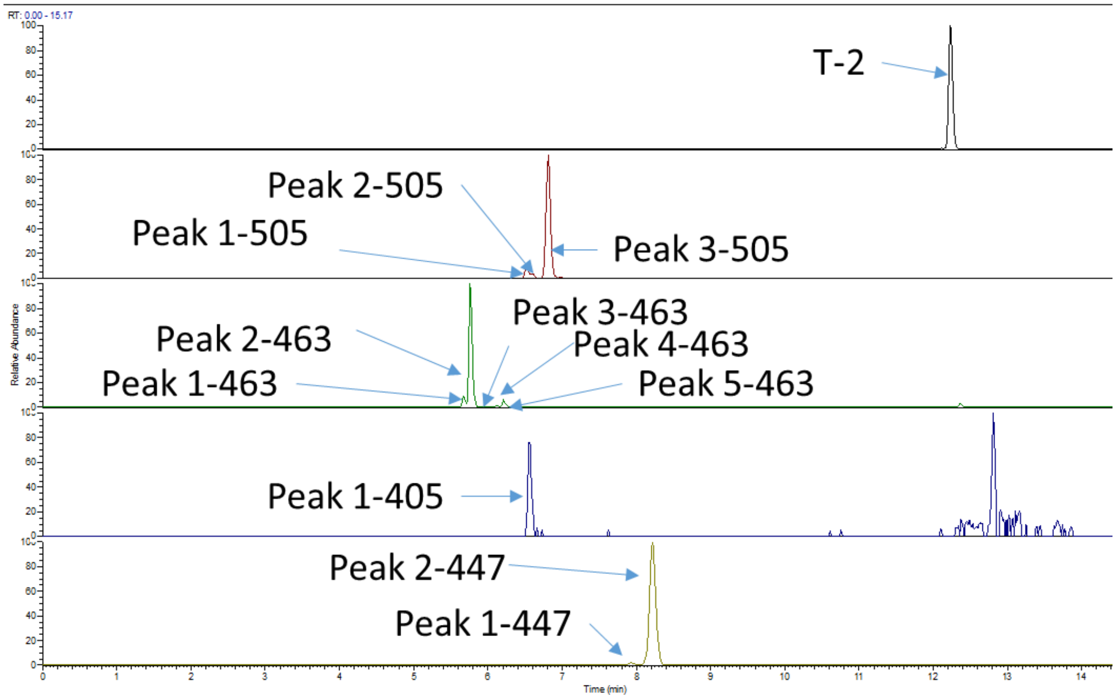

Figure S1. Extracted ion chromatogram of T-2 and its metabolites (505.2044 m/z, peak 1-505 to peak 3-505, 463.1939 m/z, peak 1-463 to peak 5-463, 405.1884 m/z, peak 1-405, 447.1989 m/z, peak 1-447 and peak 2-448) in ESI(+), detected as $[M+Na]^+$ ions.

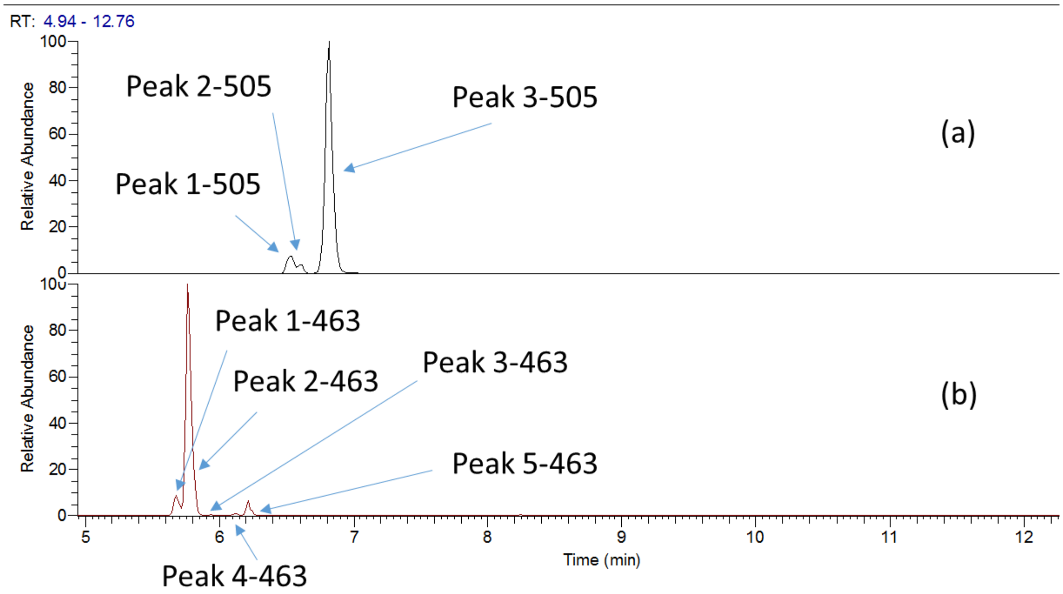

Figure S2. Zooming into extracted ion chromatogram of T-2 (505.2044 m/z) and HT-2 (463.1938 m/z) hydroxy metabolites in ESI(+), detected [M+Na]+ ions. Panel (a) shows T-2 hydroxyl metabolites, panel (b) shows HT-2 hydroxyl metabolites.

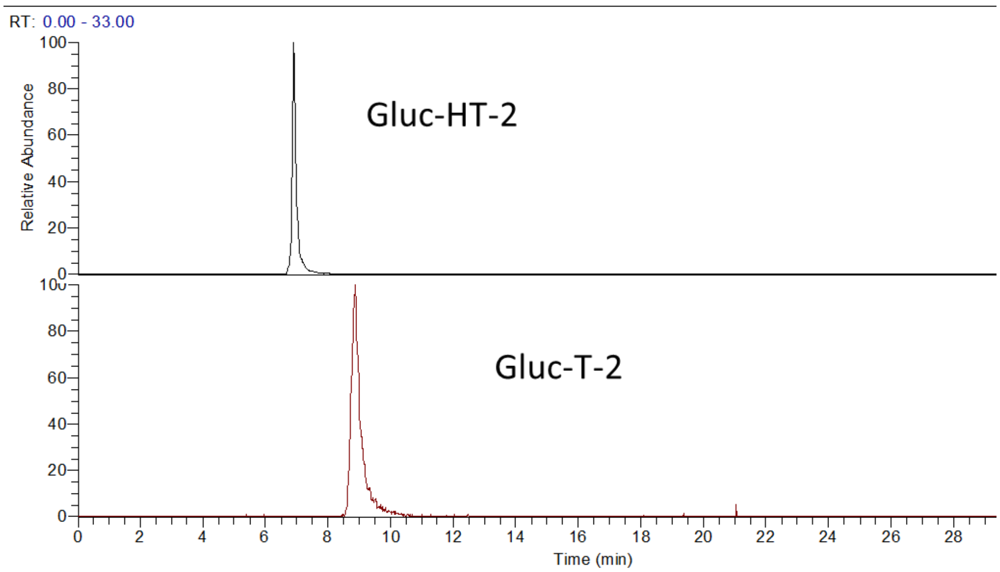

Figure S3. Extracted ion chromatogram of T-2 and HT-2 glucuronides (665.2416 m/z, Gluc-T-2, and 623.2310 m/z, Gluc-HT-2) in ESI(+), detected as[M+Na]+ ions.

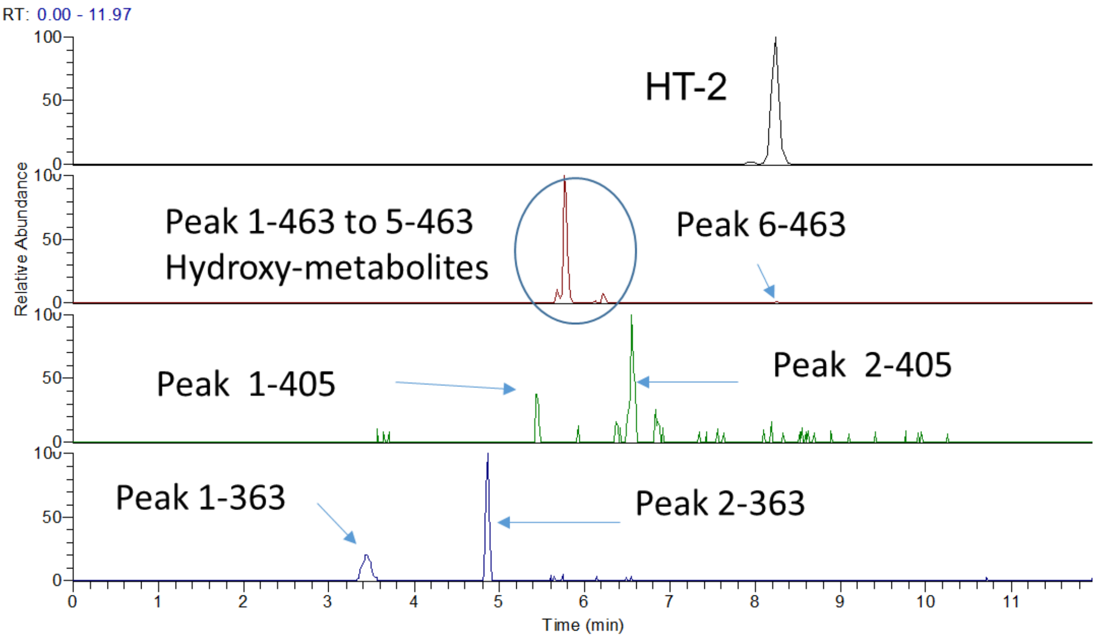

Figure S4. Extracted ion chromatogram of HT-2 and its metabolites (463.1939 m/z, peak 1-463 to peak 5-463, 405.1884 m/z, peak 1-405 and peak 2-405, 363.1414 m/z, peak 1-363 and peak 2-363) in ESI(+), detected as [M+Na]+ ions.

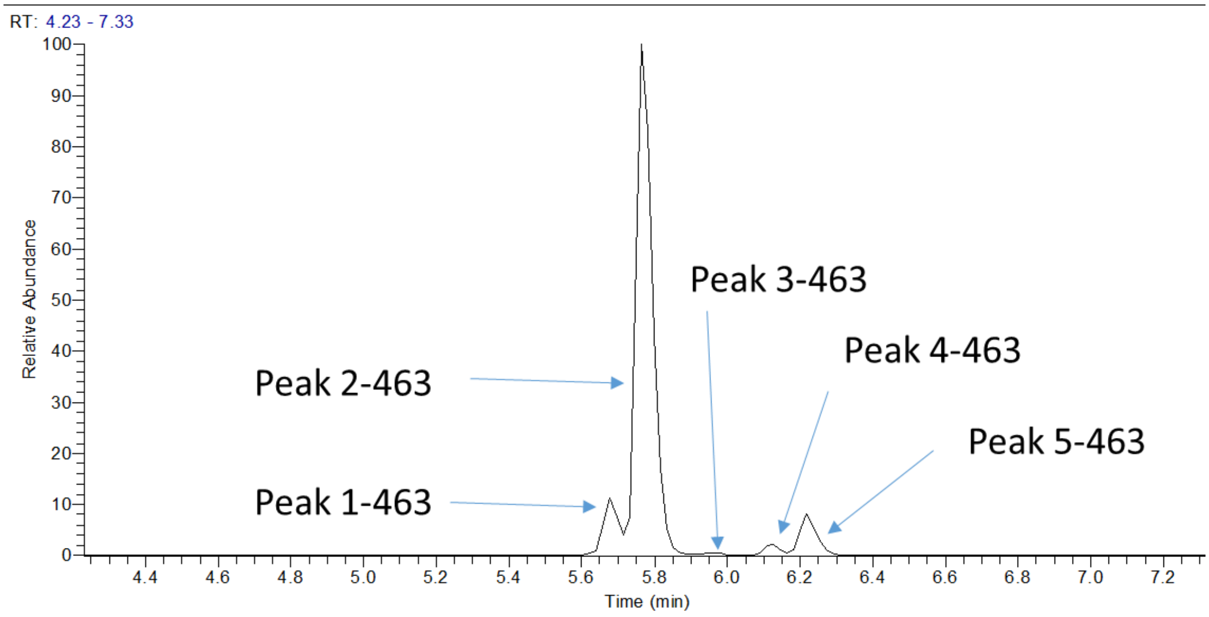

Figure S5. Zooming into extracted ion chromatogram of HT-2 hydroxyl-metabolites (463.1939 m/z, peak 1-463 to 5-463).

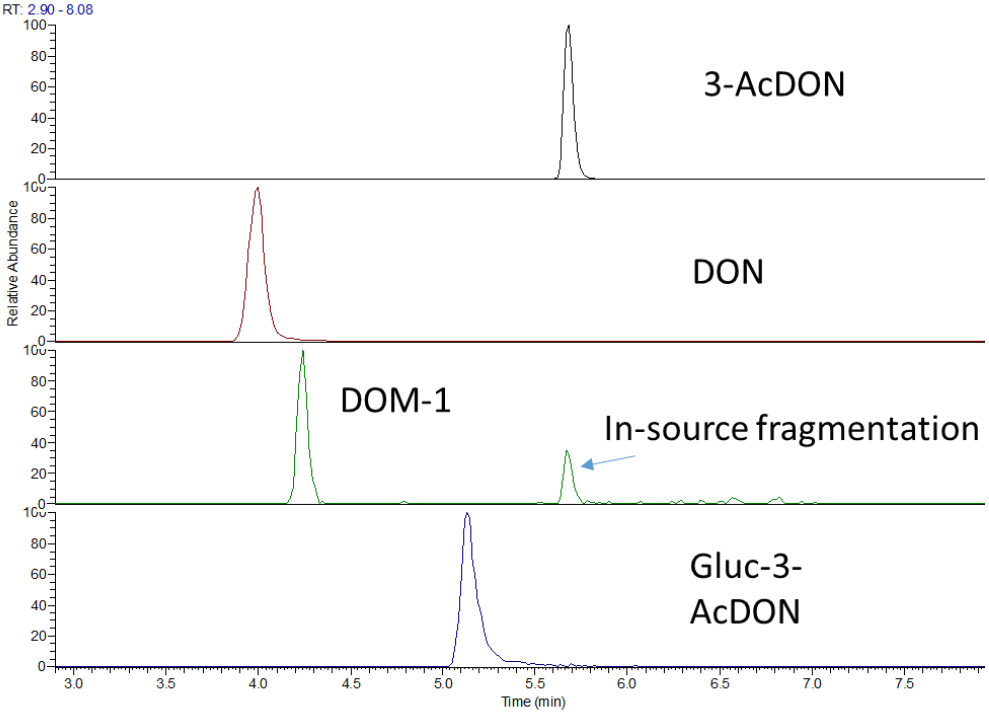

Figure S6. Extracted ion chromatogram of 3-AcDON (397.1505 m/z) and its metabolites (355.1399 m/z, DON, 339.1448 m/z, DOM-1, and 513.1613 m/z, Gluc-3-AcDON) in ESI(-), detected as [M+CH3COO-H]− ions for all except Gluc-3AcDON ([M-H]-).

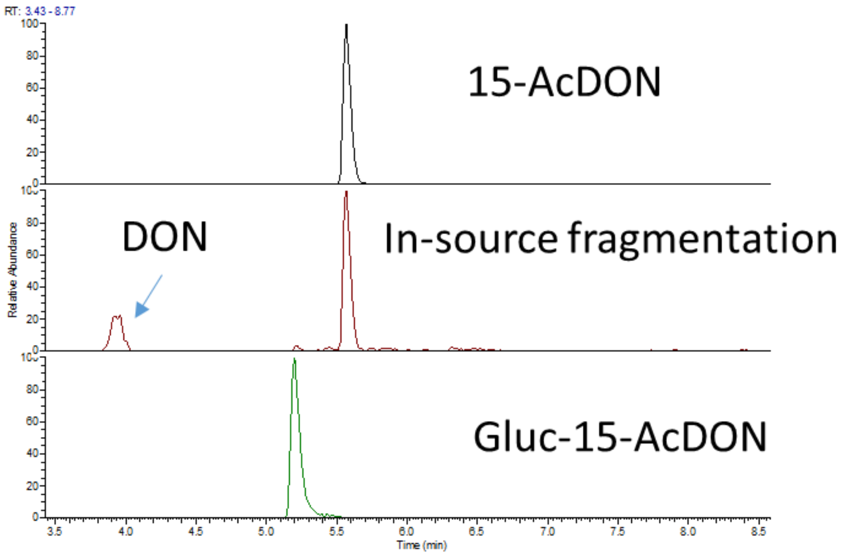

Figure S7. Extracted ion chromatogram of 15-AcDON (361.1258 m/z) and its metabolites (297.1333 m/z, DON, and 537.1579 m/z, Gluc-15-AcDON) in ESI(+), detected as [M+Na]+ ions.

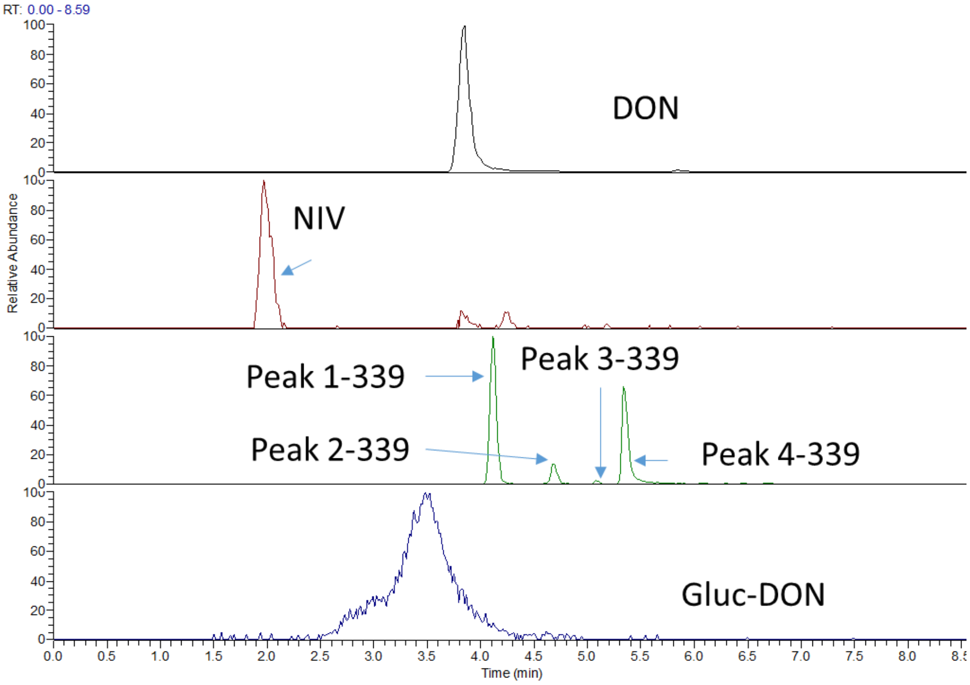

Figure S8. Extracted ion chromatogram of DON and its metabolites (371.1348 m/z, NIV, 339.1449 m/z, peak 1-339 to peak 4-339, and 471.1508 m/z, Gluc-DON) in ESI(-), detected as [M+CH3COO-H]− ions for all except Gluc-DON ([M-H]-).

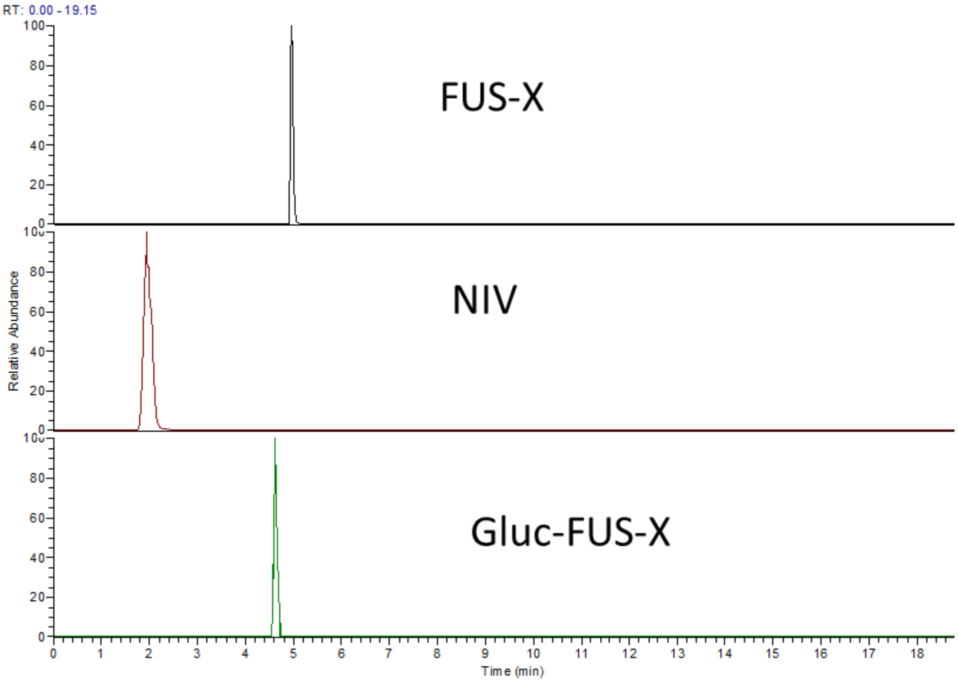

Figure S9. Extracted ion chromatogram of FUS-X (413.1454 m/z) and its metabolites (371.1348 m/z, NIV, and 529.1563 m/z, Gluc-FUS-X) in ESI(-), detected as [M+CH3COO-H]− ions for all except Gluc-FUS-X ([M-H]-).

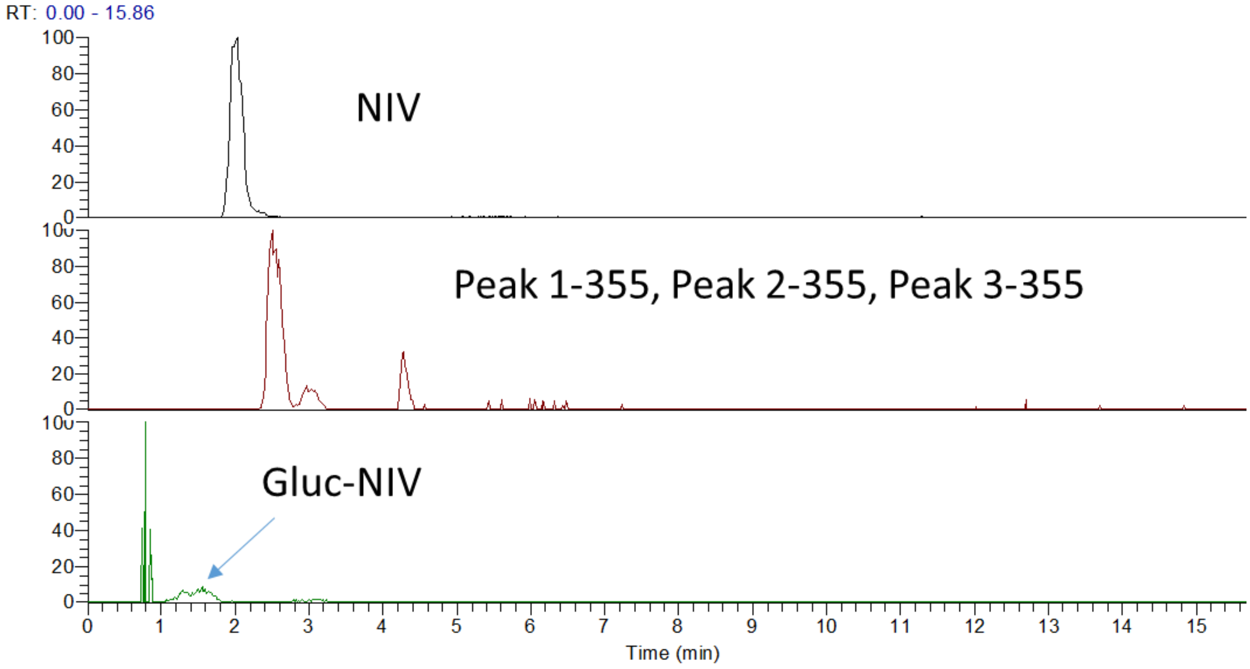

Figure S10. Extracted ion chromatogram of NIV, de-epoxy-metabolite and its isomers (355.1398 m/z, peak 1-355, peak 2-355, peak 3-355) and its glucuronides (487.1457 m/z) in ESI(-), NIV and de-epoxy-metabolite were detected as [M+CH3COO-H]− ions and glucuronides as [M-H]- ion.

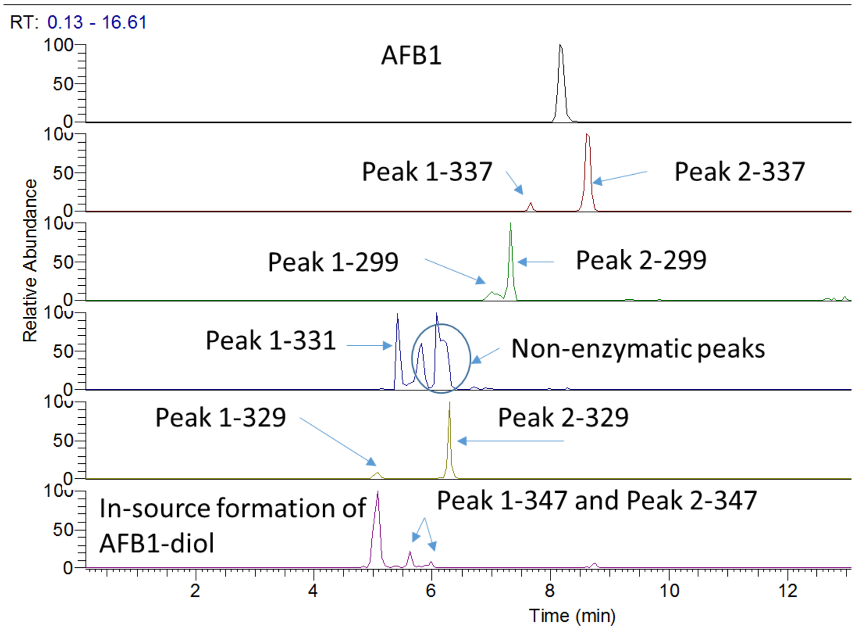

Figure S11. Chromatographic separation of AFB1 metabolites generated in phase I reactions. Extracted ion chromatogram of AFB1 (313.0707 m/z), 337.0682 m/z (peak 1-337 and peak 2-337), 299.0550 m/z (peak 1-299 and peak 2-299), 331.0812 m/z (peak 1-331), 329.0661 m/z (peak 1-329, AFBO, and peak 2-329, AFM1), 347.0761 m/z (peak 1-347 and peak 2-347) detected in ESI(+).

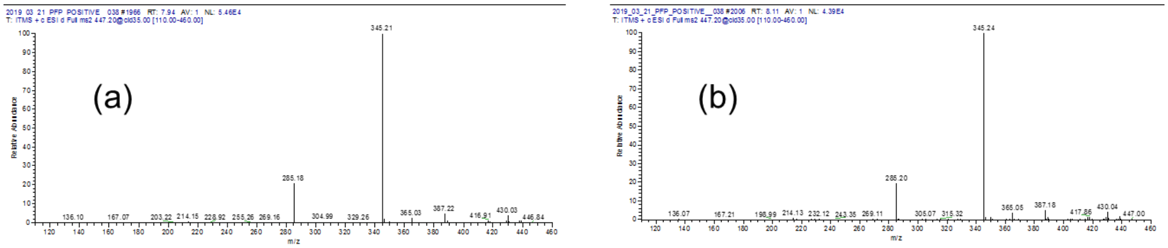

Figure S12. Product ion mass spectra of the peak 1-447 (a) which was tentatively identified as 15-deacetyl-T-2 and the peak 2-447 which was identified as HT-2, detected in ESI(+), as [M+Na]+ ions.

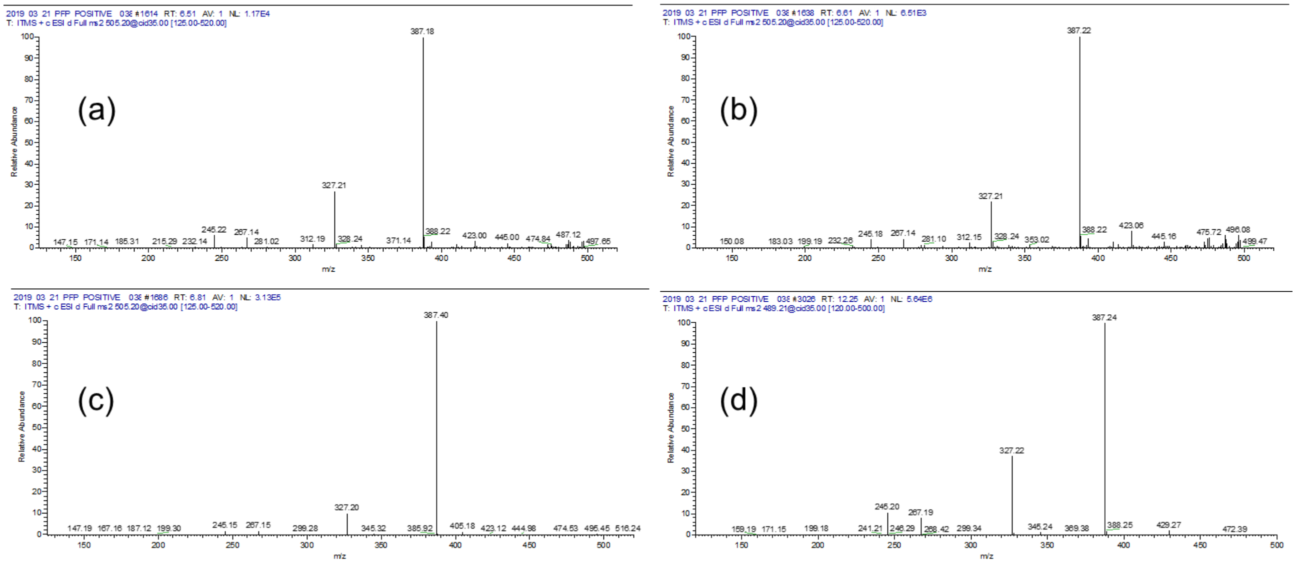

Figure S13. Product ion spectra of T-2 hydroxy metabolites at 505.2044 m/z, peak 1-505 (a), peak 2-505 (b), peak 3-505 (c) and at 489.2095, T-2, detected in ESI(+), as [M+Na]+ ions.

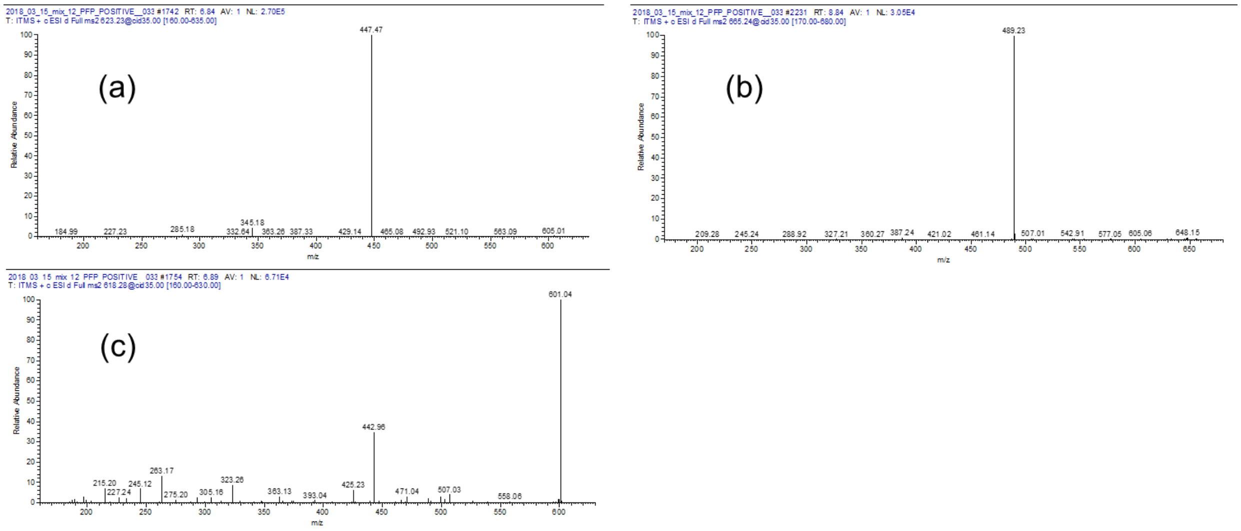

Figure S14. Product ion spectra of HT-2 glucuronide (a) at 623.2310 m/z; T-2 glucuronide (b) at 665.2416 m/zHT-2 glucuronide (c) at 618.2756 m/z, detected in ESI(+), as [M+NH4]+ions.

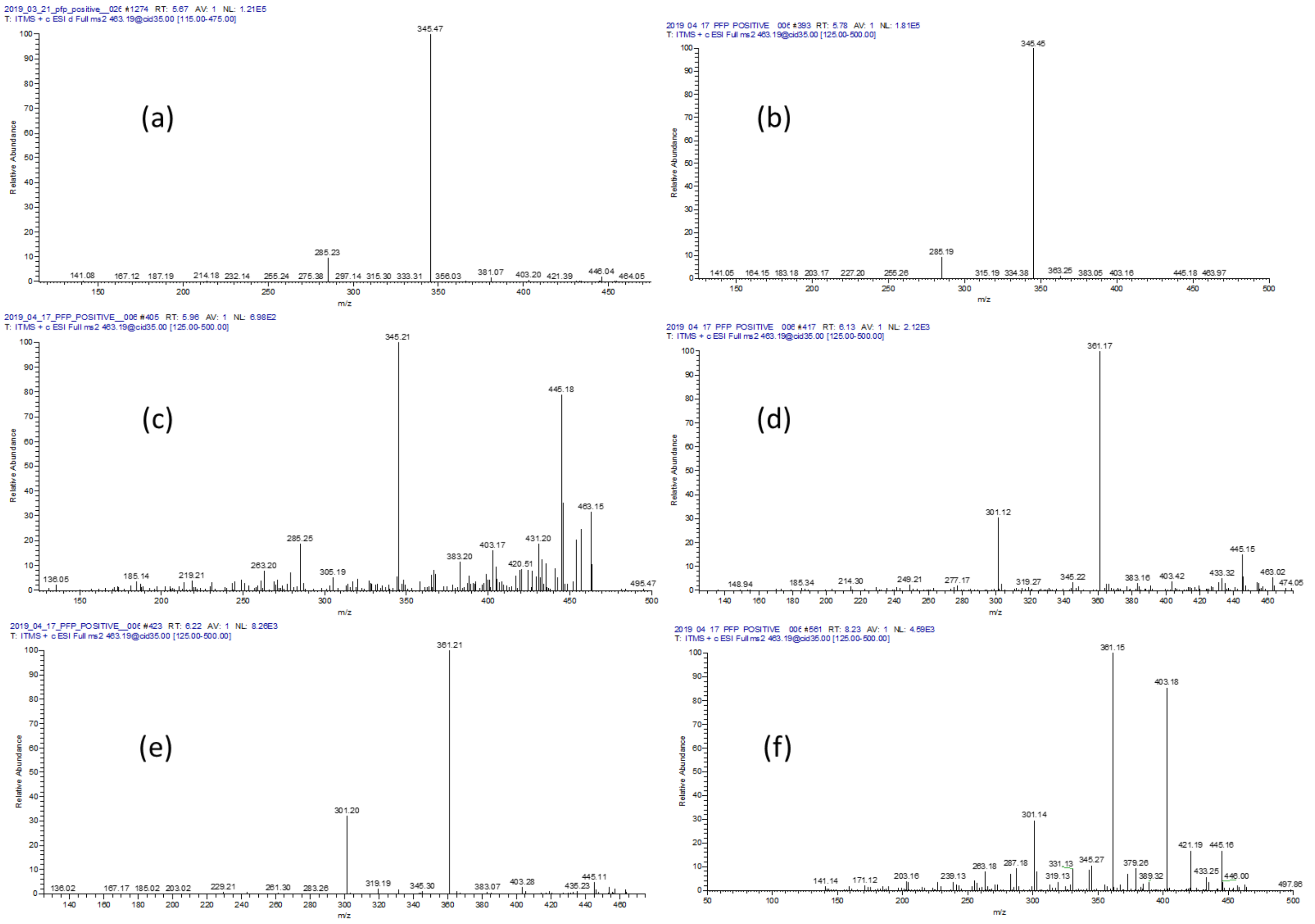

Figure S15. Product ion mass spectra of HT-2 hydroxy metabolites at 463. 1939 m/z: peak 1-463(a), peak 2-463 (b), peak 3-463 (c), peak 4-463 (d), peak 5-463 (e), peak 6-463 (f), detected in ESI(+), as [M+Na]+ions.

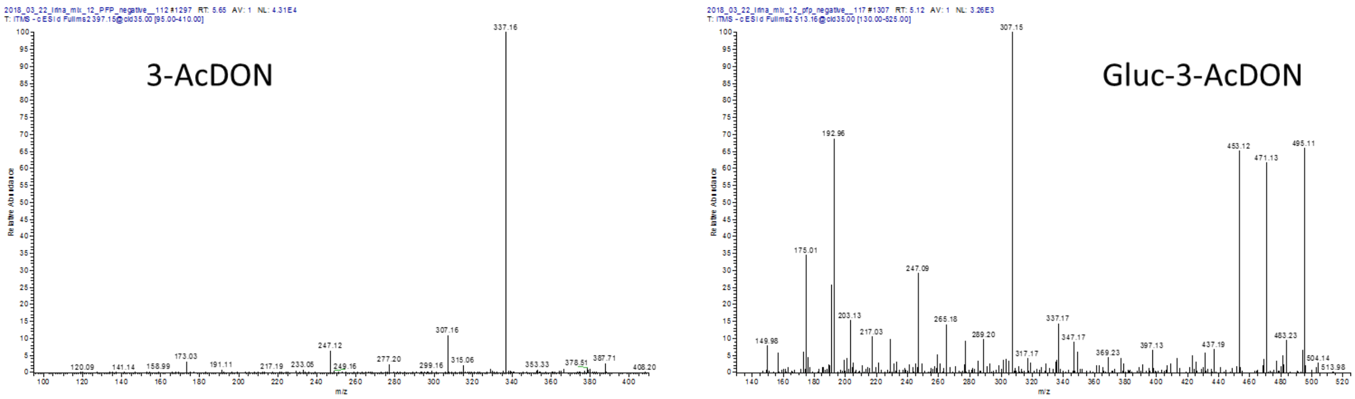

Figure S16. Product ion mass spectra of 3-AcDON at 397.1505 m/z and its glucuronide at 513.1613 m/z, detected in ESI(-), as [M+CH3COO-H]− and Gluc-3AcDON ([M-H]-ions, respectively. The other metabolites of 3-AcDON, including DON and de-epoxy-deoxynivalenol is shown in the Figure S 18 and S19, respectively.

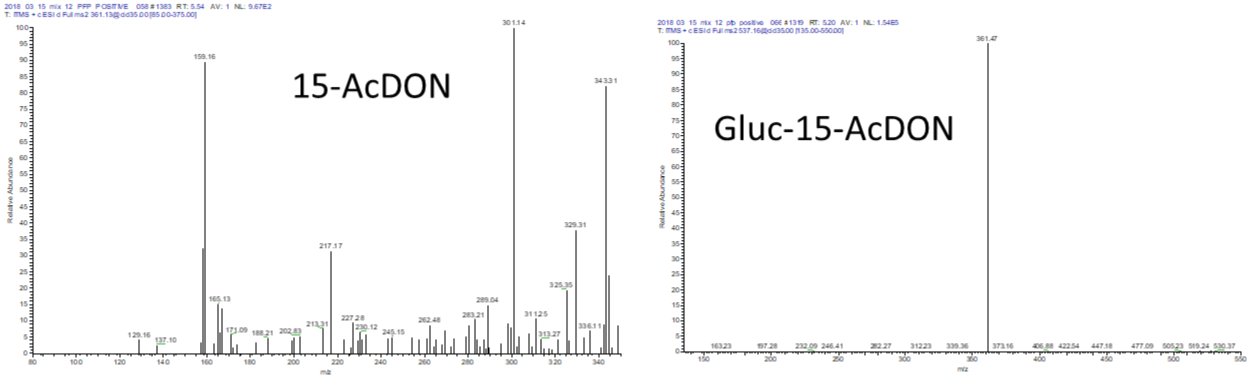

Figure S17. Product mass spectra of 15-AcDON (361.1258 m/z) and its glucuronide (537.1579 m/z), detected in ESI(+) as [M+Na]+ ions.

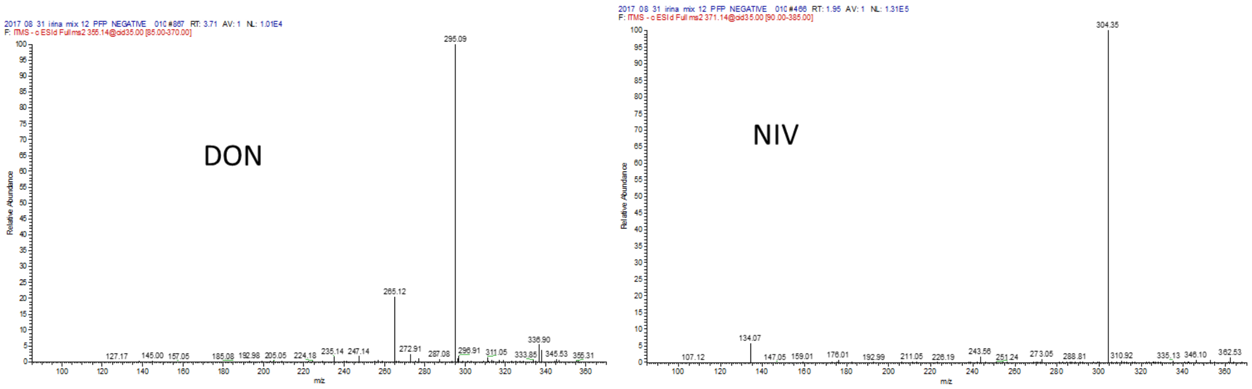

Figure S18. Product mass spectra of DON (355.1399 m/z) and NIV (371.1348 m/z) detected in ESI(-), as [M+CH3COO-H]− ions.

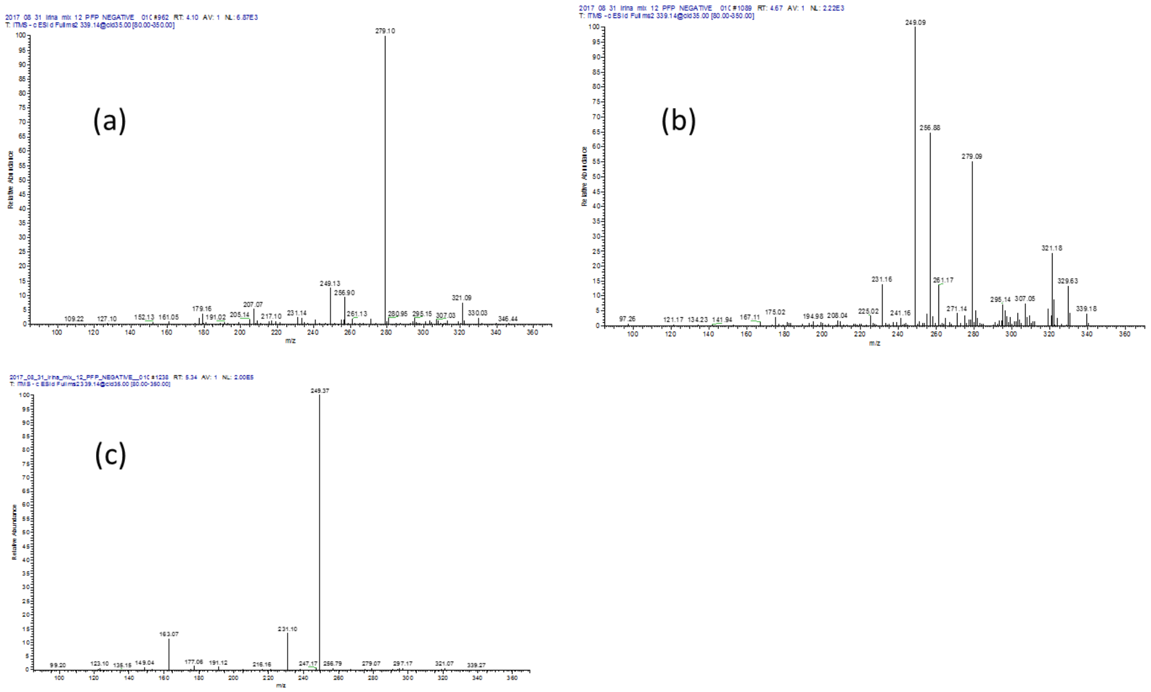

Figure S19. Product ion mass spectra of de-epoxy-deoxynivalenol at 339.1348 m/z, detected in ESI(-), as [M+CH3COO-H]− ions. Peak 1-339 (a) was observed as phase I metabolite of DON and 3-AcDON, peak 2-339 (b) and peak 3-339 (c) were observed as phase I metabolite of DON only.

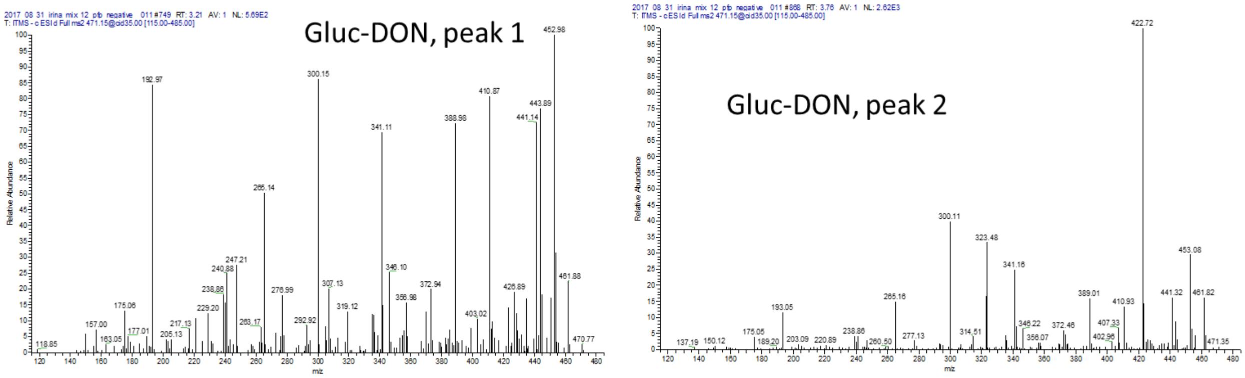

Figure S20. Product mass spectra of DON glucuronides, 471.1508 m/z, detected in ESI(-), as [M-H]− ions.

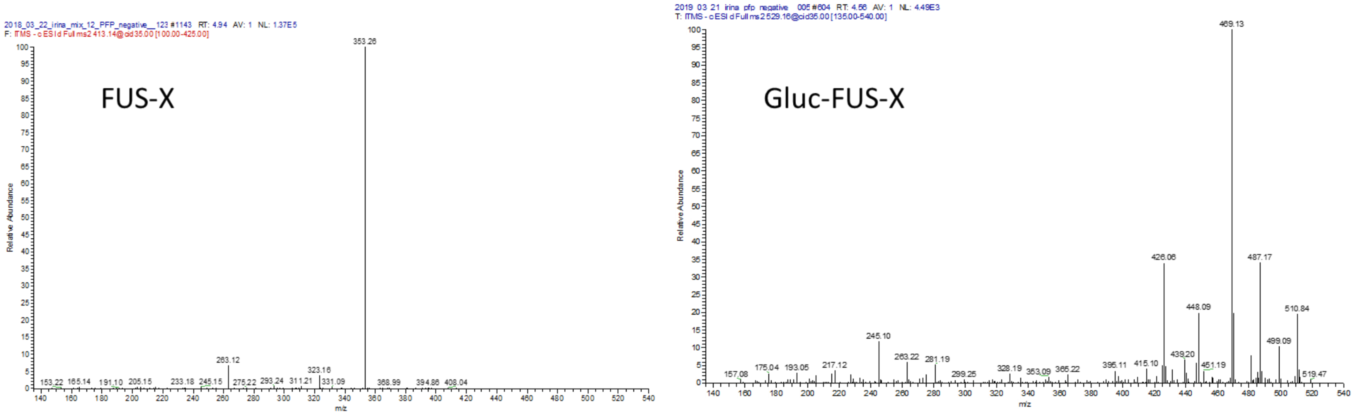

Figure S21. Product ion mass spectra of FUS-X at 413.1454 m/z and its glucuronide at 529.1563 m/z, detected in ESI(-), as [M+CH3COO-H]− and Gluc-3AcDON ([M-H]- ions, respectively. NIV mass spectrum which also was one of FUS-X metabolite was shown in the Figure S18.

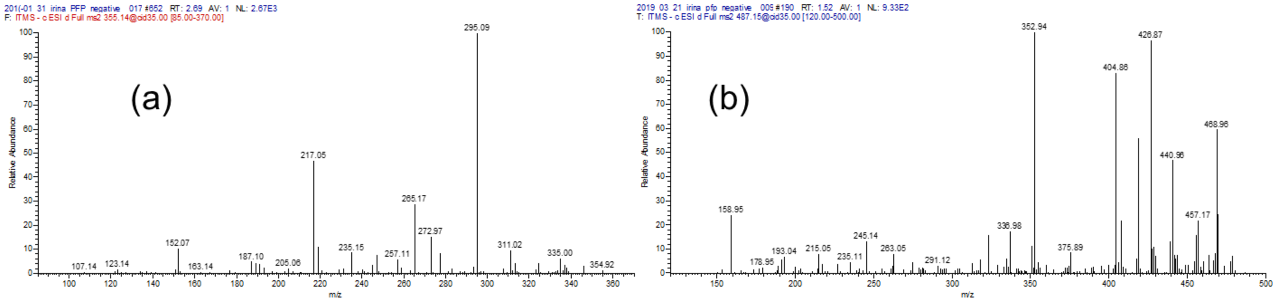

Figure S22. Product ion mass spectra of NIV metabolites: de-epoxy-nivalenol, peak 1-355 (a) at 355.1398 m/z and NIV glucuronide (b) at 487.1458 m/z, detected in ESI(-), as [M-H]- and [M+CH3COO-H]− ions, respectively. NIV mass spectrum was shown in the Figure S 18.

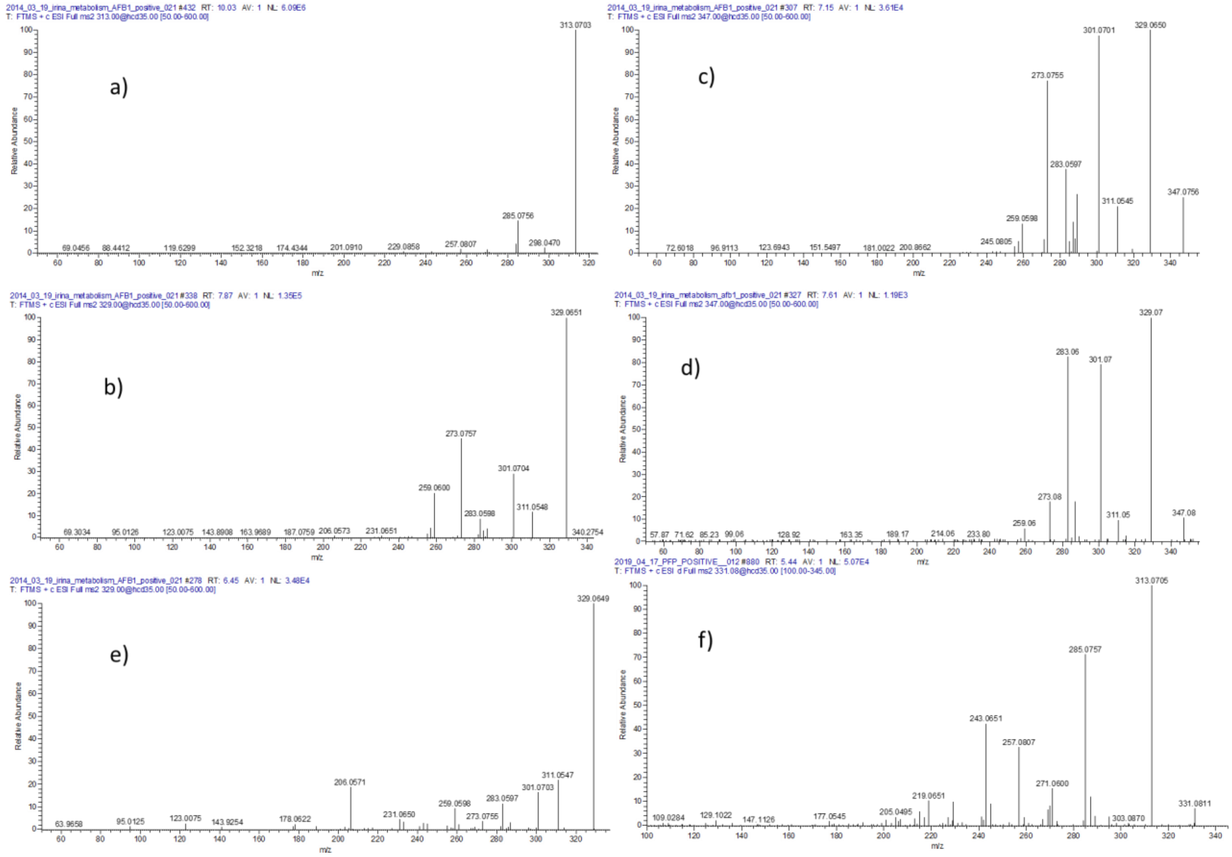

Figure S23. Product mass spectra of AFB1 (a); AFM1, peak 2-329 (b); AFB-diol, peak 1 (c); AFB-diol, peak 2 (d); AFB-8,9-endo/exo-epoxide (AFBO), peak 1-329 (e); peak 1-331 (f), detected in ESI(+), as [M+H]+ ions.

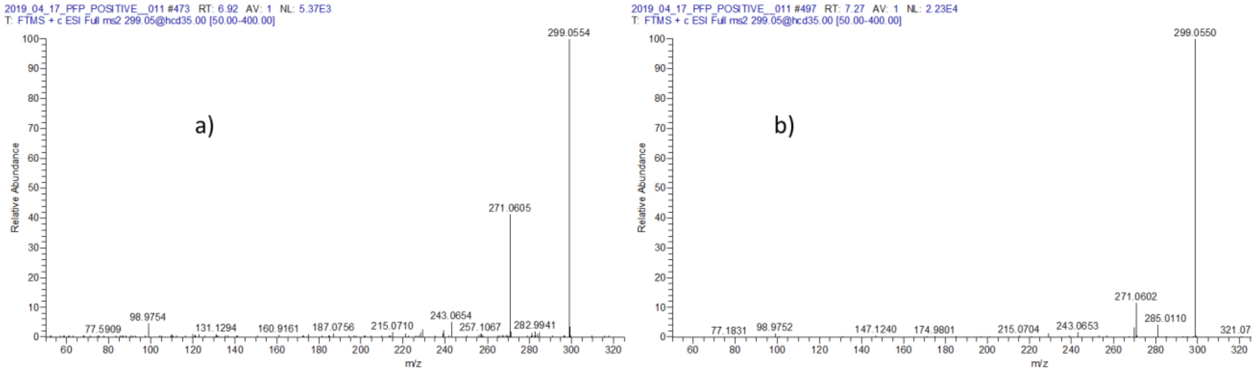

Figure S24. Product mass spectra of peak 1-299 (a) and peak 2-299 (b), detected in ESI(+), as [M+H]+ ions, identified as AFP1 and its isomer.

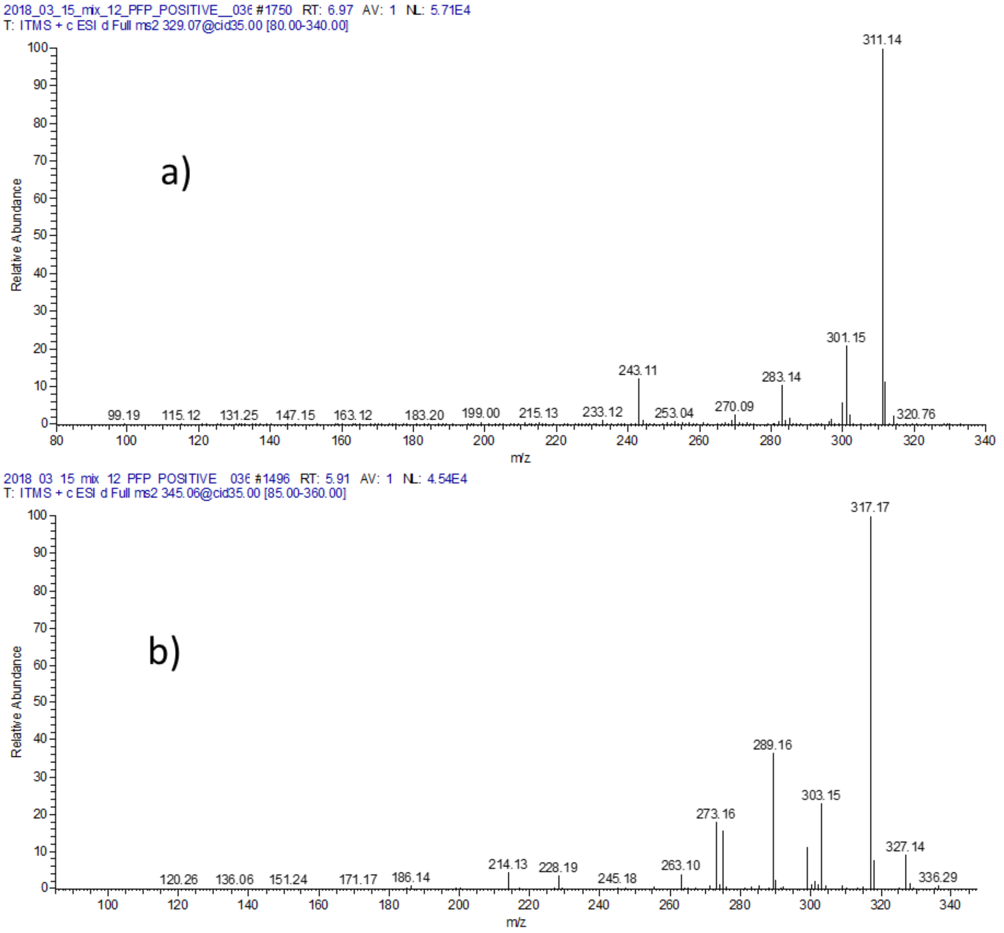

Figure S25. Product mass spectra of AFG1 (a) at 329.0656 m/z and its hydroxyl metabolite peak 1-345 (b), detected in ESI(+), as [M+H]+ ions.

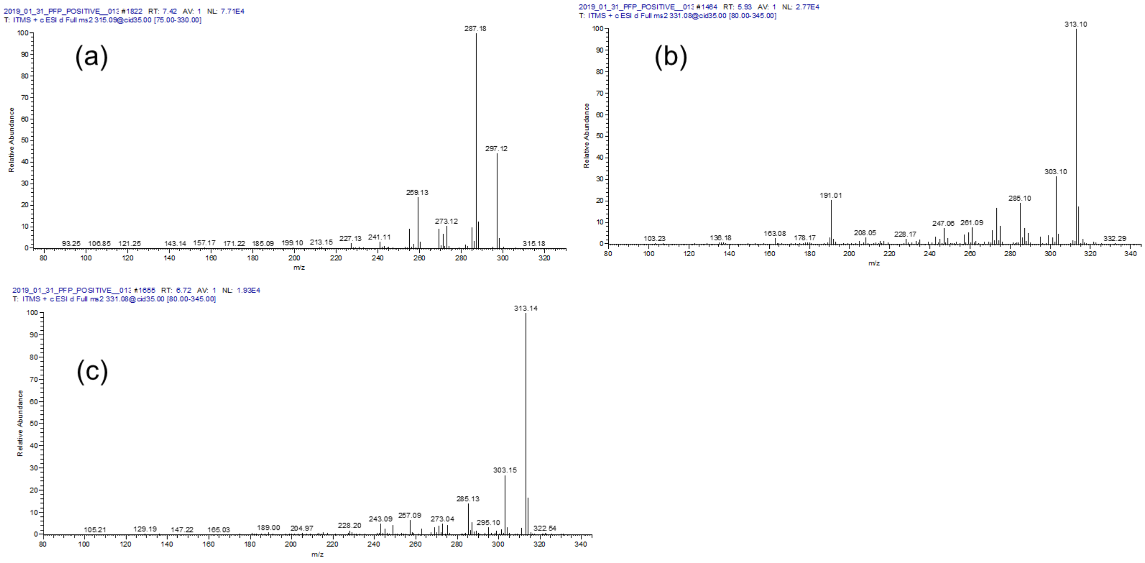

Figure S26. Product mass spectra of AFB2 (a) at 315.0863 m/z and its hydroxyl metabolites at 331.0813, peak 2-331 (b) and peak 1-331 (c), detected in ESI(+), as [M+H]+ ions.

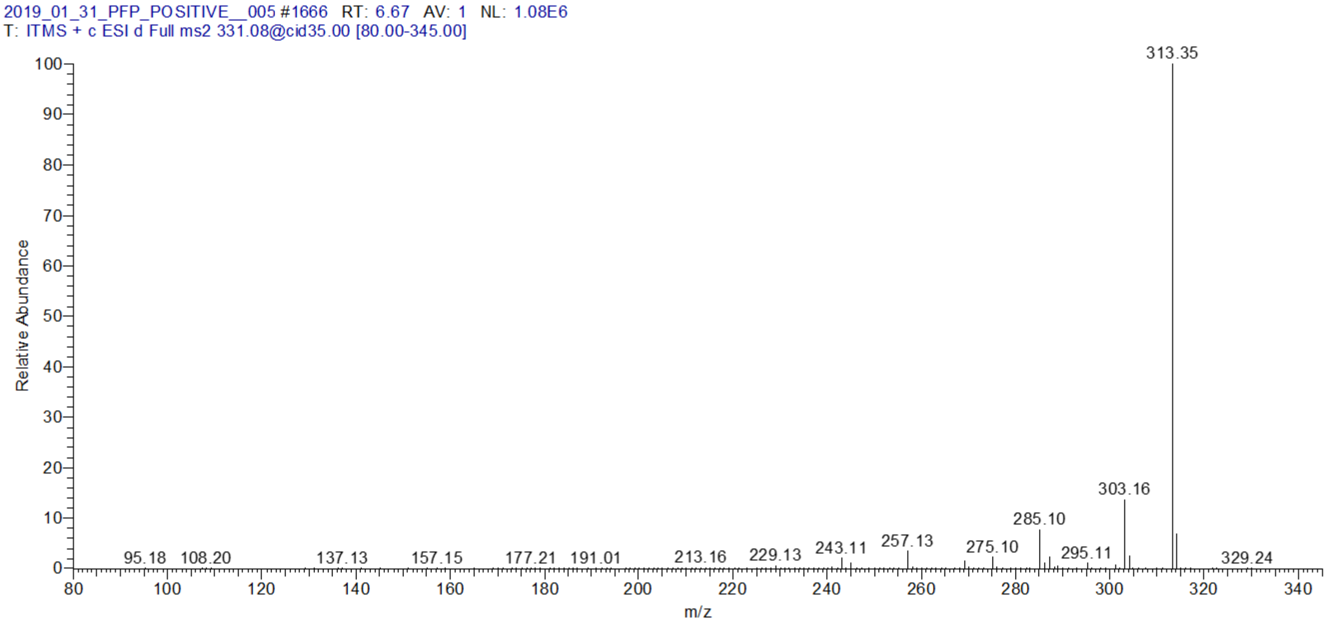

Figure S27. Product mass spectra of AFG2 at 331.0813 m/z, detected in ESI(+), as [M+H]+ ion.
